# Supplementary material for: YAP phosphorylation within integrin adhesions: Insights from a computational model
Source: Biophys J. 2024 Sep 3;123(21):3658–68. doi: 10.1016/j.bpj.2024.09.002 (PMC11560305; doi:10.1016/j.bpj.2024.09.002)
Supplement: Document S1. Figures S1–S10 and Tables S1–S5 [file mmc1.pdf]

Biophysical Journal, Volume *123*

Supplemental information

YAP phosphorylation within integrin adhesions: Insights from a computational model

Hamidreza Jafarinia, Lidan Shi, Haguy Wolfenson, and Aurélie Carlier

Supplemental information

Supplemental figures

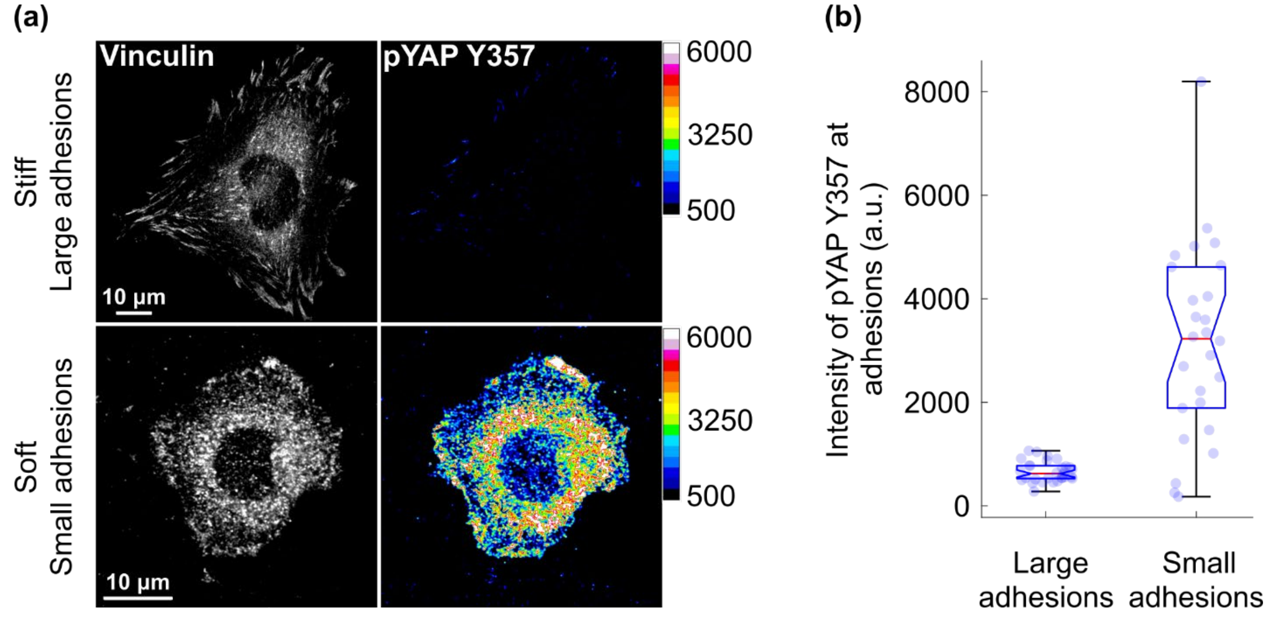

Figure S1: Experimental results for the intensity of pYAP at adhesions

(a) Representative confocal microscopy images for MEF cells that were seeded on fibronectin-coated silicon gels for 3 hours and immunostained for vinculin (grayscale, left) and pYAP Y357 (color coded for intensity, right) on stiff (top) and soft (bottom) substrates. Vinculin is a marker for adhesions. (b) Quantification of pYAP Y357 intensity within small and large adhesions in cells (N = 30 cells in each case) from the experiment shown in panel (a). Red lines in the boxplots are the median values; the bottom and top edges of the blue box represent the 25th and 75th percentiles, respectively; and the black top and bottom represent the minimal and maximal values that are not outliers, respectively. Data points are represented as blue scattered dots. Each data point represents the average value of a cell.

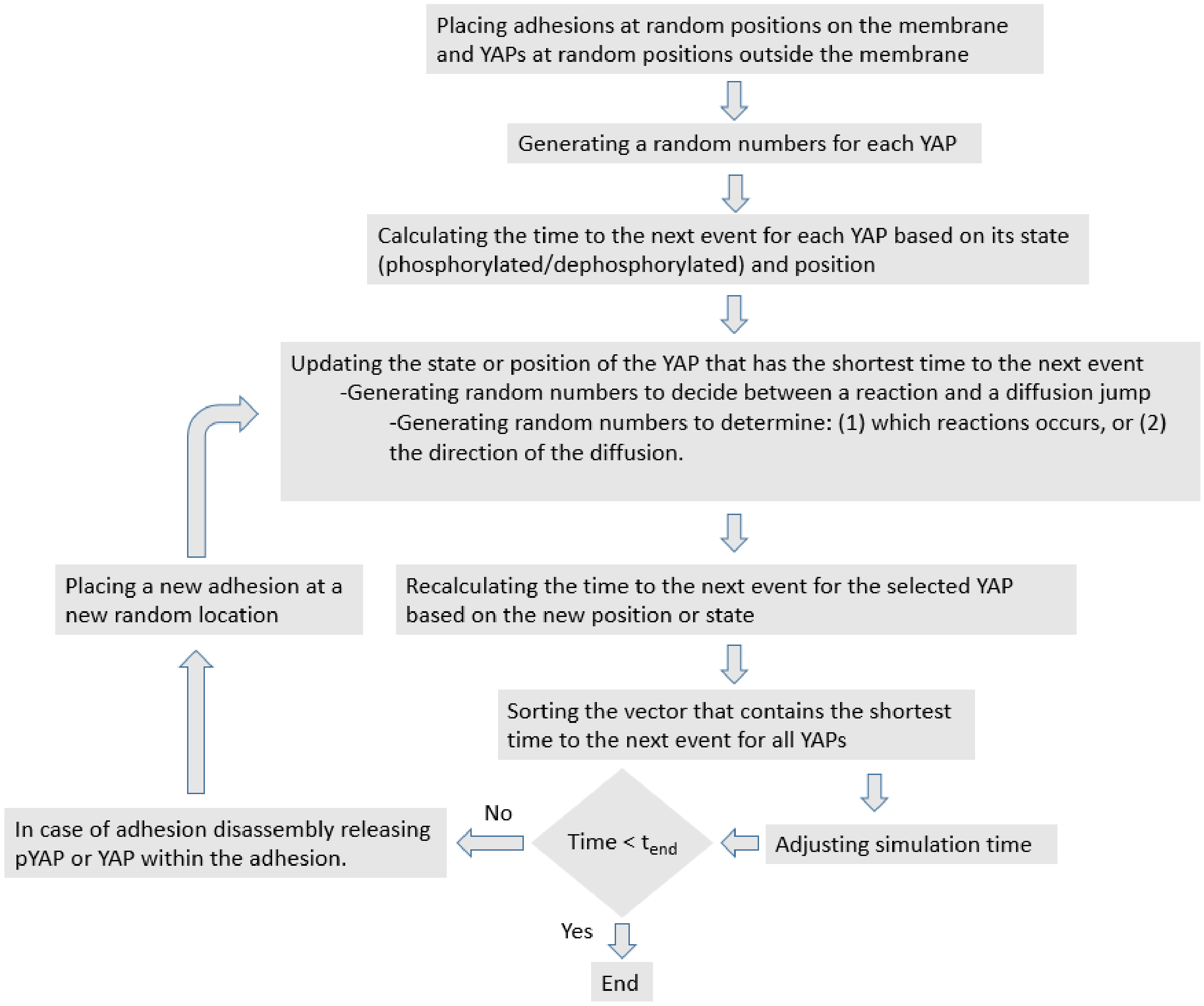

Figure S2: Simulation algorithm

See the methods section for more details. The value of t_{end} is larger than the time it takes for pYAP to reach steady state condition.

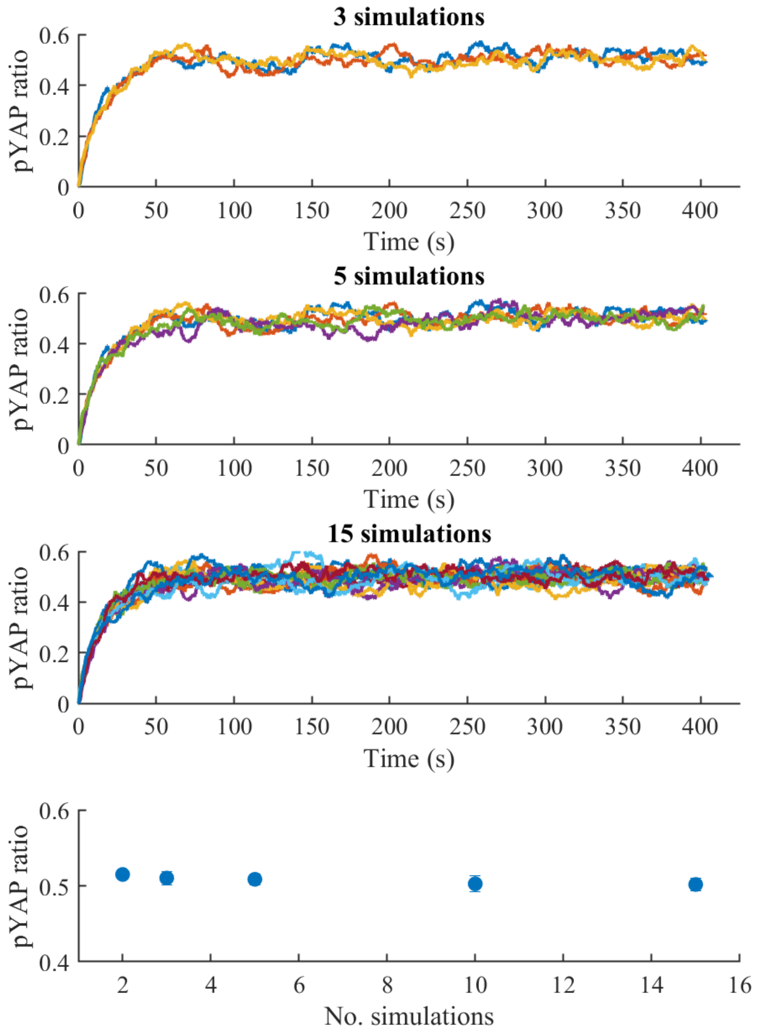

Figure S3: The average pYAP level calculated for different number of replicates

(Top panels) Temporal evolution of pYAP ratio for a set of 5, 10, and 15 simulations (Bottom panel) average pYAP ratio plotted against the number of replicates for nine small adhesions ($N = 9$), $D = 0.8\ \mu m^2/s$. For the full parameter set see Table S1.

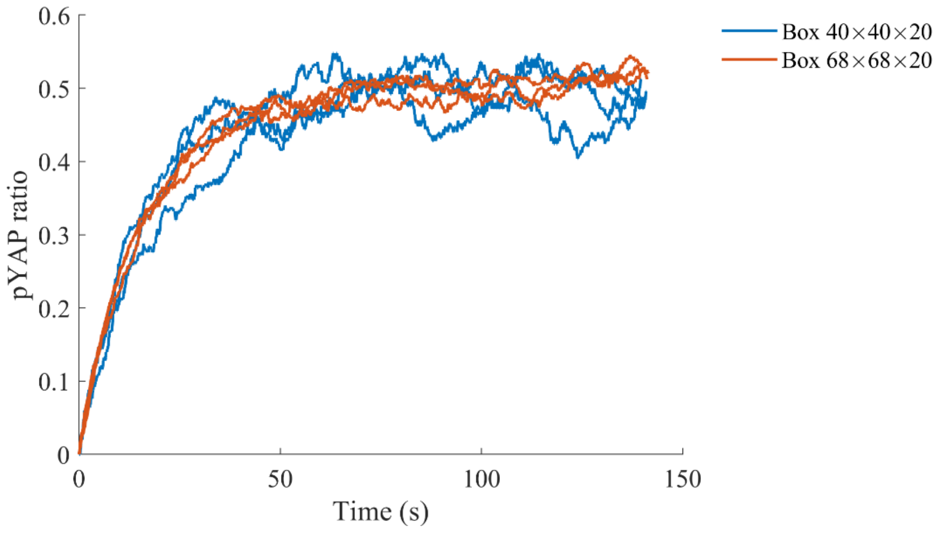

Figure S4: Comparing pYAP ratio for simulations in a larger box

Comparing pYAP ratio for simulations conducted in a larger box, 68x68x20, with 26 small adhesions (each size 3x3) and 722 YAP, with our previous simulations in a box of 40x40x20, with 9 small adhesions (each size 3x3) and 250 YAP. In both simulations the same percentage of the membrane is covered by adhesions. Additionally, the YAP concentration and the number of YAP per adhesion site are the same in both simulations. Note that the box size is not extended in z-direction to avoid changing the ratio of YAP count per adhesion site while keeping the YAP concentration constant. In these simulations $D = 0.8\,\mu m^2/s$ (for other parameters see table S1).

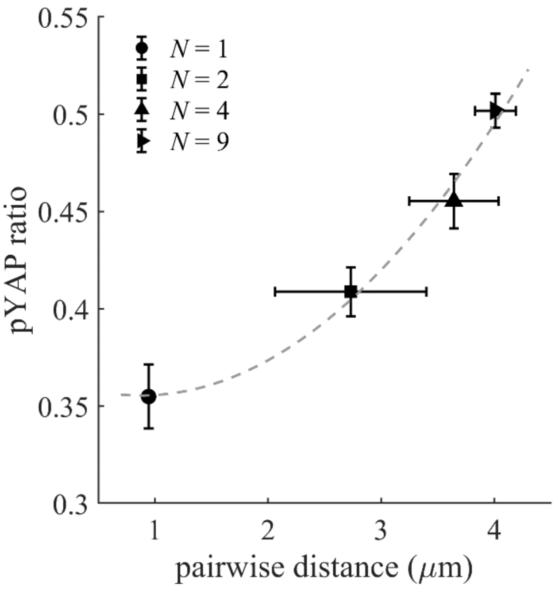

Figure S5: pYAP ratio plotted against the average pairwise distance of integrin adhesions binding sites for $N = 1, 2, 4, 9$ adhesions that all occupy the same surface area on the membrane

A second-order polynomial ($0.0143x^2 - 0.0250x + 0.3664$) is used to fit the dataset. The vertical and horizontal error bars are standard deviations calculated from 15 simulations each with a different random positions of adhesions. For a single adhesion $N = 1$, the pairwise distance remains the same across all simulations.

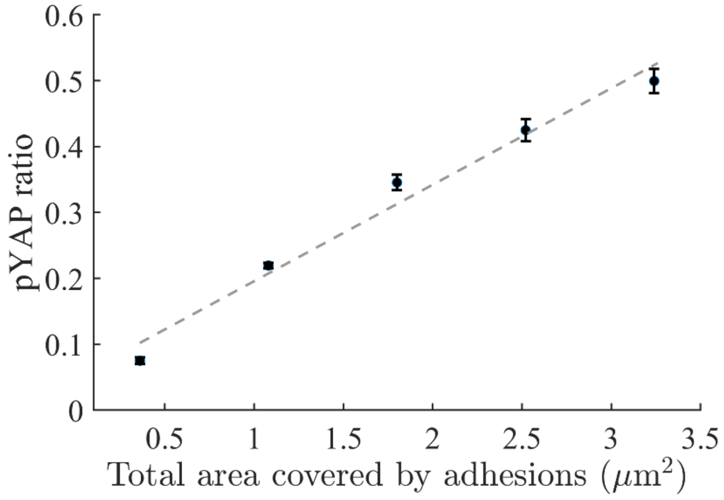

Figure S6: pYAP ratio plotted against the total area covered by adhesions

Data points correspond to 1, 3, 5, 7, and 9 small adhesions (each with a size of 3x3 or 0.36 μm^2). A linear fit ($0.1464x + 0.0494$) is used to fit the dataset. The error bars are standard deviations calculated from three simulations each with a different random positions of adhesions. The diffusion rate is set to $D = 0.8\ \mu m^2/s$ (for other parameters see table S1).

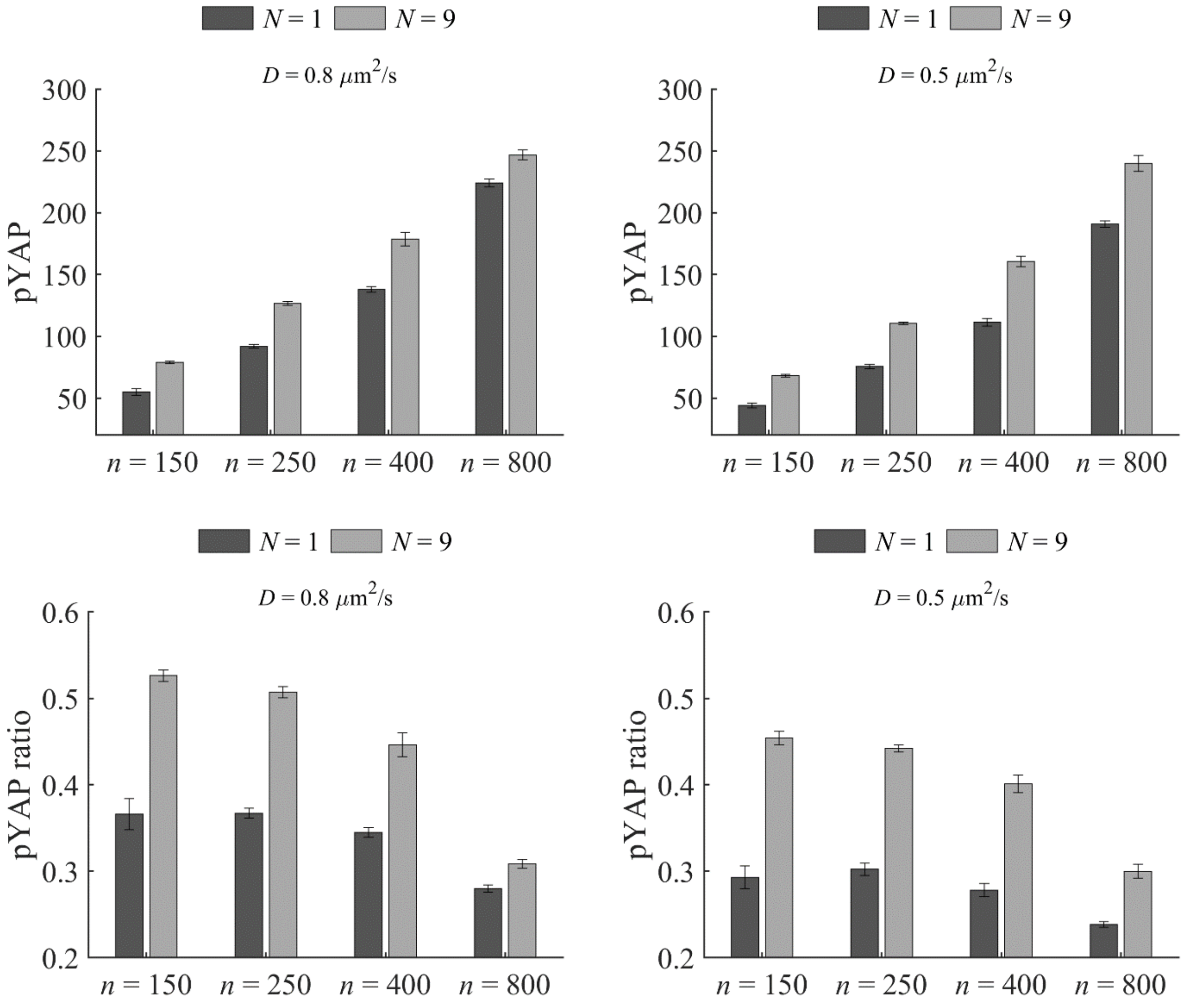

Figure S7: pYAP level plotted for different concentrations of total YAP

(Top row) pYAP level for $N = 1$ large adhesion and $N = 9$ small adhesions with the same total area at different total counts of YAP, $n_{YAP} = 150, 250, 400, 800$. Increasing the YAP concentration reduces the effect of adhesion spatial distribution on pYAP level. Reducing the diffusion rate increases the effect of the spatial distribution of adhesions on the pYAP ratio (see for example the results for $n_{YAP} = 800$ for $D = 0.8\ and\ 0.5\ \mu m^2/s$). (Bottom row) The pYAP ratio, defined as pYAP divided by the total YAP, decreases as YAP concentration increases. This reduction is due to a higher number of YAP per adhesion site and limited available binding sites for YAP at higher concentrations. For other parameters see table S1.

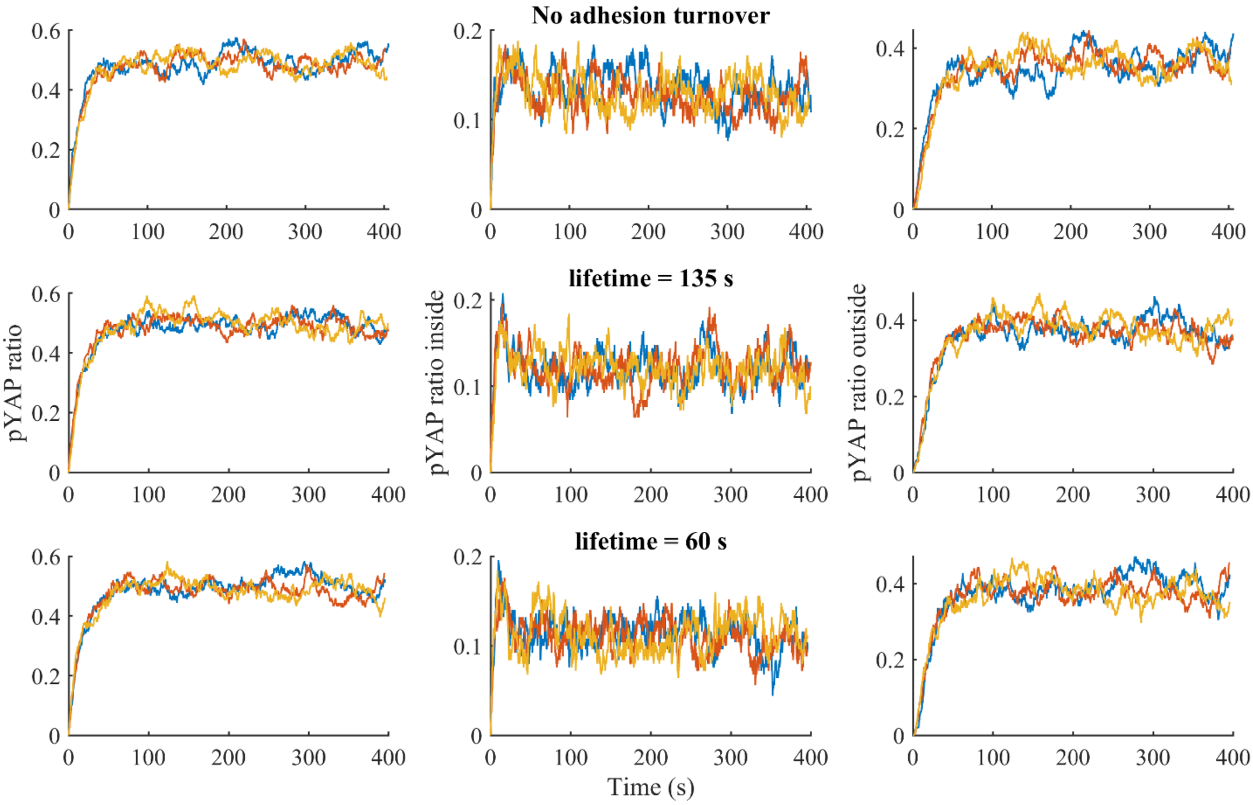

Figure S8*:* Temporal evolution of total pYAP ratio, pYAP ratio within the adhesions, and pYAP ratio outside the adhesions for nine small adhesions $N = 9$ using $R_{u,pYAP} = 0.1\ s^{-1}$, $R_{deph} = 0.035\ s^{-1}$: (Top row) no adhesion turnover, (Middle row) lifetime of 135 s, and (Bottom row) lifetime of 60 s*.* For other model parameters see table S3.

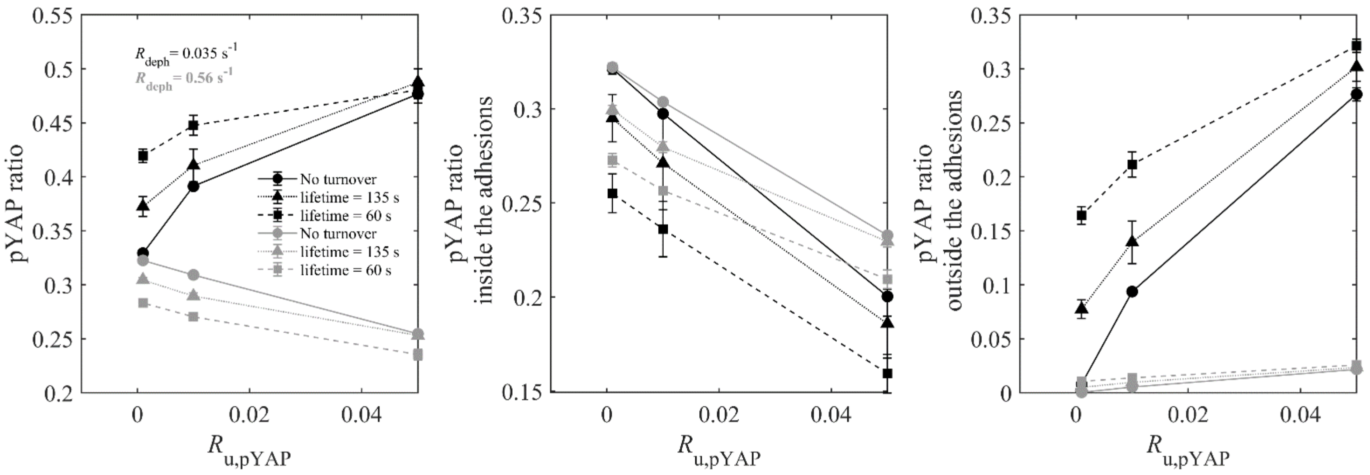

Figure S9*:* Zoomed-in panels from fig. 4 for $R_{u,pYAP} = 0.001 - 0.05\ s^{-1}$.

The total pYAP ratio, pYAP ratio within the adhesions, and pYAP ratio outside the adhesions for nine small adhesions $N = 9$ for three cases: no adhesion turnover, and lifetimes of 60 and 135 seconds for

two different dephosphorylation rates: $R_{dep} = 0.035\,s^{-1}$ (similar to Figs. 2,3) (black), and $R_{deph} = 0.56\,s^{-1}$ (gray). For the full parameter set see table S3.

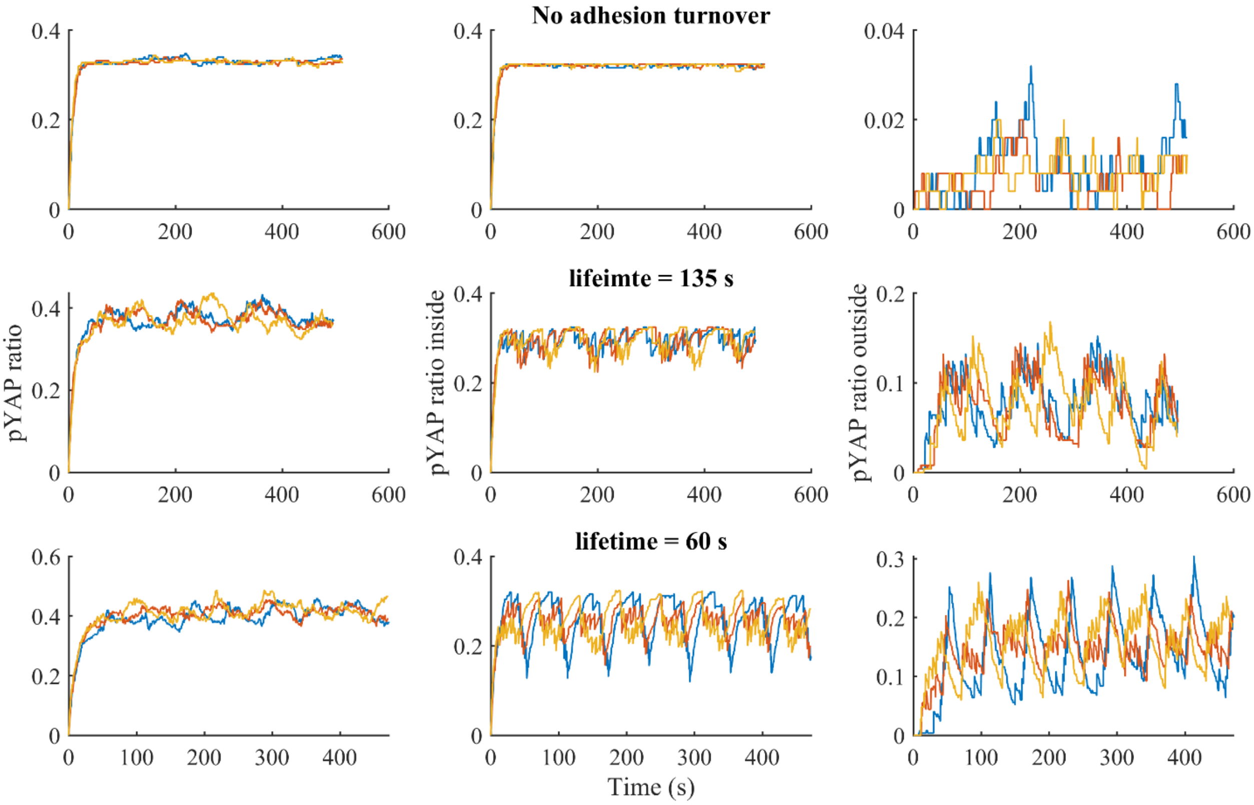

Figure S10: Temporal evolution of total pYAP ratio, pYAP ratio within the adhesions, and pYAP ratio outside the adhesions for nine small adhesions $N = 9$ using $R_{u,pYAP} = 0.001\,s^{-1}$, $R_{deph} = 0.035\,s^{-1}$: (Top row) no adhesion turnover, (Middle row) lifetime of 135 s, and (Bottom row) lifetime of 60 s. For other model parameters see table S3

Supplemental tables

Table S1: Parameter set and model variables used in Fig. 2.

Diffusion rate	D	0.8- 19 $\mu m^2/s$
YAP-adhesion binding rate	R_b	50 s^{-1}
YAP phosphorylation rate (after binding to the adhesion)	R_p	200 s^{-1}
YAP-adhesion unbinding rate	$R_{u,YAP}$	0.1 s^{-1}
pYAP-adhesion unbinding rate	$R_{u,pYAP}$	0.1 s^{-1}
Dephosphorylation rate	R_{deph}	0.035 s^{-1}
Node spacing	l	0.2 μm
Number of YAP	n_{YAP}	250
Number of adhesions	N	1,2,4,9
Lifetime	lifetime	N.A.
Number of binding sites	Number of binding sites	81
Adhesion size	Adhesion size	0.36-3.24 μm^2

Table S2: Parameter set and model variables used in Fig. 3.

Diffusion rate	D	0.8 $\mu m^2/s$
YAP-adhesion binding rate	R_b	5-100 s^{-1}
YAP phosphorylation rate (after binding to the adhesion)	R_p	200 s^{-1}
YAP-adhesion unbinding rate	$R_{u,YAP}$	0.1-0.2 s^{-1}
pYAP-adhesion unbinding rate	$R_{u,pYAP}$	0.1 s^{-1}
Dephosphorylation rate	R_{deph}	0.035 s^{-1}
Node spacing	l	0.2 μm
Number of YAP	n_{YAP}	250
Number of adhesions	N	1,9
Lifetime	lifetime	N.A.
Number of binding sites	Number of binding sites	81
Adhesion size	Adhesion size	0.36-3.24 μm^2

Table S3: Parameter set and model variables used in Fig. 4.

Diffusion rate	D	0.8 $\mu m^2/s$
YAP-adhesion binding rate	R_b	50 s^{-1}
YAP phosphorylation rate (after binding to the adhesion)	R_p	200 s^{-1}
YAP-adhesion unbinding rate	$R_{u,YAP}$	0.1 s^{-1}
pYAP-adhesion unbinding rate	$R_{u,pYAP}$	0.001-0.2 s^{-1}
Dephosphorylation rate	R_{deph}	0.035-0.56 s^{-1}
Node spacing	l	0.2 μm
Number of YAP	n_{YAP}	250
Number of adhesions	N	9
Lifetime	lifetime	60 and 135 s
Number of binding sites	Number of binding sites	81
Adhesion size	Adhesion size	0.36 μm^2

Table S4: Significance analysis for Fig. 2c and Fig. 2d. To calculate p-values we used two-sample t-test in MATLAB.

Figure 2c		Figure 2d	
Pairs	p-value	Pairs ($D = 0.8\ \mu m^2/s$)	p-value
Cases 1, 2	0.1004	$N = 1,2$	0.0148
Cases 1, 3	0.0154	$N = 1,4$	0.0006
Cases 1, 4	0.0006	$N = 1,9$	0.0006
Cases 2, 3	0.0423	$N = 2,4$	0.0007
Cases 2, 4	0.0008	$N = 2,9$	0.0011
Cases 3, 4	0.0330	$N = 4,9$	0.0141

Table S5: Fitting parameters for Fig. 3 (bottom panel).

Fitting function: $pYAP = k1R_b/(k2 + R_b)$		
Cases	$k1$	$k2$
$N = 1, D = 0.8\ \mu m^2/s$	101.3	5.4
$N = 9, D = 0.8\ \mu m^2/s$	143.7	7.1
$N = 1, D = 19\ \mu m^2/s$	210.6	12.1
$N = 9, D = 19\ \mu m^2/s$	213.7	11
