# Supplementary material for: YAP phosphorylation within integrin adhesions: Insights from a computational model
Source: Biophys J. 2024 Sep 3;123(21):3658–68. doi: 10.1016/j.bpj.2024.09.002 (PMC11560305; doi:10.1016/j.bpj.2024.09.002)
Supplement: Document S2. Article plus supporting material [file mmc2.pdf]

Biophysical Journal
Article

YAP phosphorylation within integrin adhesions: Insights from a computational model

Hamidreza Jafarinia,[1] Lidan Shi,[2] Haguy Wolfenson,[2] and Aurélie Carlier[1,*]
[1]MERLN Institute for Technology-Inspired Regenerative Medicine, Department of Cell Biology-Inspired Tissue Engineering, Maastricht University, Maastricht, the Netherlands and [2]Department of Genetics and Developmental Biology, Rappaport Faculty of Medicine, Technion – Israel Institute of Technology, Haifa, Israel

ABSTRACT Mechanical and biochemical cues intricately activate Yes-associated protein (YAP), which is pivotal for the cellular responses to these stimuli. Recent findings reveal an unexplored role of YAP in influencing the apoptotic process. It has been shown that, on soft matrices, YAP is recruited to small adhesions, phosphorylated at Y357, and translocated into the nucleus triggering apoptosis. Interestingly, YAP Y357 phosphorylation is significantly reduced in larger mature focal adhesions on stiff matrices. Building upon these novel insights, we have developed a stochastic model to delve deeper into the complex dynamics of YAP phosphorylation within integrin adhesions. Our findings emphasize several key points: firstly, increasing the cytosolic diffusion rate of YAP correlates with higher levels of phosphorylated YAP (pYAP); secondly, increasing the number of binding sites and distributing them across the membrane surface, mimicking smaller adhesions, leads to higher pYAP levels, particularly at lower diffusion rates. Moreover, we show that the binding and release rate of YAP to adhesions as well as adhesion lifetimes significantly influence the size effect of adhesion-induced YAP phosphorylation. The results highlight the complex and dynamic interplay between adhesion lifetime, the rate of pYAP unbinding from adhesions, and dephosphorylation rates, collectively shaping overall pYAP levels. In summary, our work advances the understanding of YAP mechanotransduction and opens avenues for experimental validation.

SIGNIFICANCE YAP plays an important mechanosignaling role in essential processes such as cell proliferation and apoptosis. Our recent study shows accumulation in small adhesions of YAP phosphorylated at the Y357 residue. In contrast, formation of larger adhesions on stiff matrices significantly reduces YAP Y357 phosphorylation. In this study, we explore the dynamic interplay between adhesion properties and YAP phosphorylation with the aim to identify different factors that could lead to the experimentally observed behavior. Our findings highlight the significance of adhesion size, spatial distribution, and lifetime, as well as the diffusion rate of YAP and the rate of YAP binding to adhesions, in modulating YAP phosphorylation levels. Overall, this work contributes to an improved understanding of YAP mechanotransduction mechanisms.

INTRODUCTION

Understanding how mechanical signals are converted into biochemical cues is a fundamental question in mechanobiology. Yes-associated protein (YAP) and its homolog transcriptional co-activator with PDZ-binding motif (TAZ) are important mechanosignaling components that play a role in regulating essential processes such as cell proliferation, differentiation, and tissue homeostasis (1,2) as well as disease progression (3,4). Various mechanical cues can trigger the activation of YAP/TAZ (4–7), although the underlying mechanisms of action are not entirely clear.

The Hippo pathway serves as the primary regulator of YAP/TAZ. This pathway is initiated upon phosphorylation of the mammalian Ste20 kinase1/2 (MST1/2) complex by various upstream signaling mechanisms including focal adhesions, adherens junctions, and cytoskeletal mechanics (reviewed in detail in (1–4,8,9)). When the Hippo signaling pathway is active, YAP and TAZ are phosphorylated (for YAP at serine 127), followed by their subsequent cytoplasmic sequestration and degradation. Conversely, when the Hippo signaling pathway is off, nonphosphorylated YAP/TAZ can enter the nucleus to regulate gene expression through association with DNA-binding transcription factors

Submitted April 26, 2024, and accepted for publication September 3, 2024.
*Correspondence: a.carlier@maastrichtuniversity.nl
Editor: Guy Genin.
https://doi.org/10.1016/j.bpj.2024.09.002
© 2024 The Author(s). Published by Elsevier Inc. on behalf of Biophysical Society.
This is an open access article under the CC BY license (http://creativecommons.org/licenses/by/4.0/).

such as TEA domain transcription factors (TEADs), which is associated with cell proliferation (10).

Notably, phosphorylation of YAP at tyrosine 357 (Y357) can be a positive trigger for YAP activity independent of the Hippo pathway. Indeed, YAP Y357 phosphorylation by Src in response to mechanical stimuli (11) or by c-Abl kinase in response to DNA damage (12) has been associated with apoptosis. In this scenario, the Y357 phosphorylated YAP (pYAP) is known to interact with p73, as opposed to the interaction with TEAD, to activate apoptosis (12). Importantly, our recent work showed that, on soft matrices, YAP is recruited into small adhesions, phosphorylated at the Y357 residue by Src/c-Abl, and translocated into the nucleus, ultimately leading to apoptosis (13). Interestingly, adhesion reinforcement and formation of larger adhesions on stiff matrices significantly reduces YAP Y357 phosphorylation (see an example of experimental results in Fig. S1), although the underlying mechanisms are not clear (13). Importantly, the accumulation of pYAP Y357 (referred to as pYAP in this study) by numerous small adhesions, as we recently reported (13), is a novel mechanism previously unexplored in the literature for YAP or any other signaling proteins and differs from the activation of signaling molecules such as FAK within focal adhesions (14) and from the focal adhesion signaling cascade that regulates YAP activation through serine 127 phosphorylation (15).

The direct recruitment of YAP to adhesions poses several intriguing questions regarding the impact of adhesion properties on the level of pYAP Y357: do adhesion size and turnover rate influence the concentration of pYAP? If so, what is the regulatory mechanism? And what is the impact of (p) YAP binding and release in the adhesions on overall pYAP levels?

To tackle these challenges, we adopted a biophysics-based computational approach, allowing us to systematically dissect different interacting mechanisms. Most computational studies to date have investigated the dynamics of adhesions and downstream signaling separately (14–19), as reviewed in (20). Models of YAP activation have focused mostly on the F-actin and myosin pathway, providing useful insights into the mechanisms of FAK activation in response to extracellular matrix, subsequent regulation of F-actin and myosin, and the resulting YAP serine 127 (de)phosphorylation and nuclear translocation (15–17,19,21). However, previous spatial models have considered adhesions as continuous rather than discrete entities on the membrane and did not study YAP Y357 phosphorylation. Consequently, they have not incorporated the spatial distribution of adhesions or the critical aspects of the Y357 phosphorylation site for downstream signaling. Furthermore, the influence of adhesion size and turnover rate has not been included in the previous models.

To overcome these limitations, we developed a novel stochastic model that incorporates YAP Y357 phosphorylation within discrete adhesions. This model is the first, to the best of our knowledge, to investigate how adhesion size, spatial distribution, and turnover impact YAP Y357 phosphorylation. We conducted sensitivity analyses of model parameters and simulated various scenarios for the release of YAP from the adhesions, thereby predicting how different conditions may affect YAP activity initiated from YAP's direct interaction with the adhesions. Our findings highlight the significance of several factors—YAP diffusion rate, adhesion size and spatial distribution, and YAP binding rate to the adhesions—in influencing overall pYAP levels, providing a theoretical foundation for the previously experimentally observed behavior. We also highlight that the dynamic interplay between adhesion lifetime, the rate of pYAP unbinding from adhesions, and dephosphorylation rates shapes pYAP levels in a nontrivial manner. Overall, the results of this study contribute to an improved understanding of YAP mechanotransduction mechanisms.

MATERIALS AND METHODS

The dynamics of YAP phosphorylation within integrin adhesions is simulated using a spatially stochastic Gillespie algorithm (22,23). Compared to the model developed for FAK phosphorylation within integrin adhesions (14), which uses dimensionless parameters, our model provides specific values for adhesion sizes, diffusion rates of (p)YAP, (un)binding rates, YAP concentration, and the dephosphorylation rate of pYAP, based on empirical data. These parameters are crucial as they significantly affect the pYAP ratio (Figs. 2, 3, and 4). Our model also integrates specific lifetimes for nascent adhesions. This allows us to explore two mechanisms for the release of (p)YAP from the adhesions. Additionally, we incorporate a dephosphorylation rate for pYAP, another aspect not addressed in (14). This inclusion enables us to examine the complex interactions between adhesion lifetime, YAP unbinding rates, and YAP dephosphorylation dynamics, as demonstrated in Fig. 4. A detailed explanation of our model is provided below.

Simulations take place within a 3D lattice of size $40 \times 40 \times 20$ grid cells in x, y, and z directions, with a node spacing of $l = 0.2\ \mu m$, representing a part of the cell. The model uses periodic boundary conditions along the x and y directions to minimize finite-size effects and closed boundaries at the top and bottom surfaces (as shown in Fig. 1). In this way, steric hindrance is modeled at the top/bottom surfaces. The plasma membrane is represented as the $z = 1$ plane at the bottom of the simulation domain. At the beginning of each simulation, N integrin adhesions are randomly positioned on the membrane with square arrangements while ensuring volume exclusion. When $N > 1$, the initial distribution ensures that adhesions are not clumped together. To further test the effect of adhesion distribution, we also consider cases where adhesions are confined to randomly chosen position within $r = 0.25$ (area size 20×20 grid cells or 16 μm^2) and $r = 0.09$ (area size 12×12 grid cells or 5.76 μm^2) of the membrane area with r representing the area of the confined region divided by the total simulated membrane area (see Fig. 2 c, right panels). In these cases, we also allow adhesions to clump together during the initial placement on the membrane.

In our model, we select the smallest and largest adhesions to cover an area of 3×3 lattice sites (equal to 0.36 μm^2) and 9×9 lattice sites (equal to 3.24 μm^2), respectively. These selections fall within the size ranges experimentally observed for nascent adhesions, which are smaller than 1 μm in diameter, and mature focal adhesions, which can be up to 10 μm^2 (24–27). Experimental evidence shows that YAP is recruited to small adhesions on soft matrices and phosphorylated at tyrosine 357 (13). In contrast, the phosphorylation levels are very low in large adhesions on stiff substrates. Moreover, slight inhibition of actomyosin contractility allows

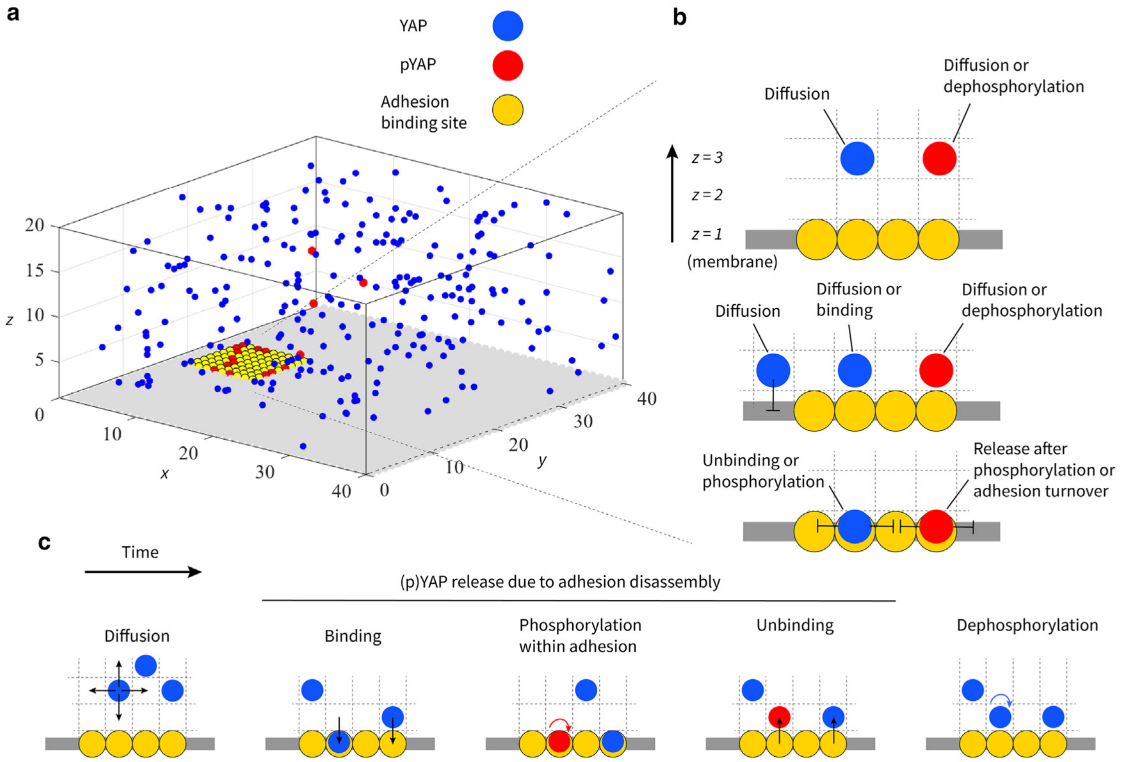

FIGURE 1 A sample snapshot of the simulation domain and schematics illustrating the simulated events. (*a*) 3D simulation domain for YAP phosphorylation within integrin adhesions. Integrin adhesions depicted with yellow beads on the membrane. Blue beads show YAP and red beads show phosphorylated YAP (pYAP). (*b*) Schematic representations of the diffusion and reaction events incorporated in the model. (*c*) Sequence of events in the model.

adhesion growth on soft matrices, leading to reduced YAP phosphorylation (13). Based on these observations, we do not explicitly model the matrix stiffness but input the adhesion size directly into our model to systematically explore scenarios that can explain the experimentally observed trends. In our model, small adhesions represent adhesions on soft substrates and large adhesions represent focal adhesions formed on stiff substrates.

We assume that YAP is dephosphorylated initially, and 250 YAPs, $n_{YAP} = 250$ (equivalent to 1.62 nM), are placed randomly in the $z > 1$ region. The mean concentration of YAP in cells has not been measured in previous studies; however, our estimate is consistent with experimental findings for the mean FAK concentration in cells, which is measured in the nanomolar range (28). We assume a constant total YAP (YAP plus pYAP) as the production and degradation timescales (∼hours) are much larger than the modeled processes (∼seconds, minutes). Experimental data indicate that the half-life of the YAP protein is approximately 6–8 h (29), which is much larger than our total simulation time (minutes). Previous mathematical models that focused on YAP/TAZ signaling have treated the total amounts of key proteins such as FAK (19,30), RhoA (19), and YAP/TAZ (15) as constants. Notably, the degradation processes in these models often do not represent a loss of protein but a transition between active and inactive states, which in our study is accounted for by the rates of (de)phosphorylation (see the following sections).

In each simulation step, YAP undergoes diffusion or reaction events (Fig. 1). Diffusion to neighboring sites is modeled as a hopping reaction with a rate d. Reaction events encompass YAP binding to adhesions, phosphorylation of YAP within adhesions, release of pYAP from adhesions, and subsequent dephosphorylation of released YAP in the cytosol (see Table 1 for an overview of the parameter values and below for more details on each of these events).

In the stochastic algorithm, each YAP is assigned a random number ($rand$) from a uniform distribution between 0 and 1. This number is used to calculate the first event time (τ_i) for each YAP: $\tau_i = -\log(rand)/(r_i + d_i)$, where r_i represents the sum of the rates of potential reaction events (i.e., binding, unbinding, phosphorylation, and dephosphorylation), and d_i stands for the rate of diffusion jumps to neighboring sites. We assume that the diffusion rate does not vary spatially; therefore, d_i does not vary for each YAP except in an adhesion where (p)YAP does not diffuse and $d_i = 0$ (see section events in the $z = 1$ region). The simulation begins by updating the position or state of the YAP with the shortest time to the next event, denoted as $t = \tau_m$. To decide between a reaction and a diffusion jump, another random number ($rand$) is generated from a uniform distribution between 0 and 1. If $rand < r_i/(r_i + d_i)$, a reaction is chosen; otherwise, a diffusion jump is selected. If a reaction is selected, then a similar approach is used to decide which reaction occurs. If a diffusion jump is selected, then another random number is generated to choose between the six neighboring sites. Depending on the chosen event, the state or position of the YAP is updated. Subsequently, the time to the next event for the YAP is recalculated based on the new position or state as $t_{new} = -\log(rand)/(r_i + d_i) + t$. The algorithm then continues by updating the position or state of the next (p)YAP with the shortest time to the next event. In cases involving adhesion turnover, if the time reaches the lifetime of a specific adhesion, that adhesion disassembles and reappears in a new random position on the membrane. We assume that the newly formed adhesion maintains the same size (26). This process accounts for the dynamic nature of adhesion turnover within the simulation. A schematic representation of the algorithm is presented in Fig. S2.

The stochastic algorithm iterates until a steady state is achieved, where the number of pYAP fluctuates around an average value. The initial pYAP number is set to be zero. In each simulation, all adhesions have the same size and the simulation is repeated three times for a total of $10^7 - 5 \times 10^7$ steps starting from new random positions of adhesions. pYAP ratio, defined as the count of pYAP divided by the total YAP count (YAP plus pYAP), is calculated, and the average of three simulations is reported. Error bars represent standard deviations calculated from at least

TABLE 1 Range of model parameters used in this study

Diffusion rate	D	0.8–19 $\mu m^2/s$ (17,31–33)
YAP-adhesion binding rate	R_b	5–100 s^{-1} (33)
YAP phosphorylation rate (after binding to the adhesion)	R_p	200 s^{-1}
YAP-adhesion unbinding rate	$R_{u,YAP}$	0.02–0.2 s^{-1} (34–37)
pYAP-adhesion unbinding rate	$R_{u,pYAP}$	0.001–0.2 s^{-1} (34–37)
Dephosphorylation rate	R_{deph}	0.035–0.56 s^{-1} (15,19)
Node spacing	l	0.2 μm
Number of YAPs	n_{YAP}	150–800 (28)
Number of adhesions	N	1, 2, 4, 9
Lifetime	lifetime	60–135 s (26,40)
Number of binding sites	number of binding sites	81
Adhesion size	adhesion size	0.36–3.24 μm^2 (24–27)

See also Tables S1–S3 in the Supporting Material for specific parameters used in each figure.

three rounds of simulations. In Fig. S3, we show that performing more simulations does not alter the average pYAP ratio. In Fig. S4 we also show that increasing the box size while maintaining the same percentage of the area covered by adhesions does not alter the model output. Simulations are conducted in MATLAB version 2022 and the code is available at https://github.com/carliercomputationallab/YAP-phosphyrylation-within-adhesion-stochastic-model. Subsequent sections outline the events occurring in various regions of the simulation domain. The model parameter settings are summarized in Table 1, and an illustrative depiction of different events in the algorithm is presented in Fig. 1.

Events in the $z > 2$ region

Within this region, both YAP and pYAP undergo diffusion through a hopping reaction characterized by a rate $d = nD/l^2$ where $D = 0.8 - 19\ \mu m^2/s$ is the diffusion rate (15,17,31–33), $l = 0.2\ \mu m$ is the node spacing, and $n = 6$ is the number of directions in which YAP can diffuse. The selected range for diffusion rates align with both previous experimental measurements for cytoplasmic diffusion of adhesion proteins such as paxillin and FAK (32,33) as well as the earlier computational models of YAP/TAZ signaling (15,31). To account for steric repulsion, movements that would place one YAP on the same lattice site as another YAP are rejected. Additionally, pYAP in this region can undergo dephosphorylation at a rate $R_{deph} = 0.035 - 0.56\ s^{-1}$, similar to dephosphorylation rates of FAK and YAP in previous computational models (15,19).

Events in the $z = 2$ region

YAP has been shown to be recruited to integrin adhesions (13), although the molecular mechanism for YAP binding to the adhesions is not completely understood. To mimic this process, in our model we assume that YAP can bind to the adhesions when it is one lattice site away from the membrane, directly above the sites occupied by the adhesion. The binding event takes place in the z direction, modeled as a jump from $z = 2$ to the adhesion with a rate $R_b = 50\ s^{-1}$. The value of R_b must be sufficiently high to align with experimental observations of YAP presence within adhesions (13). However, at high binding rates (see Fig. 3, bottom panel), pYAP ratio reaches a plateau, indicating that further increases in the binding rate no longer significantly impact the pYAP ratio. Therefore, we selected a binding rate of $R_b = 50\ s^{-1}$ for our baseline model, which approximates the point at which this plateau occurs. This choice of R_b is also consistent with the dynamics of paxillin binding that occurs with the kinetics of ~20 ms (33).

In our model, we assume that only unphosphorylated YAP can bind to the adhesion site, and binding to sites already occupied within the adhesion is not allowed. Similar to the $z > 2$ region, within $z = 2$ region, the diffusion of YAP and diffusion and dephosphorylation of pYAP are possible events.

Events in the $z = 1$ region

Upon binding, YAP can be phosphorylated at a rate R_p within the adhesion. Alternatively, YAP can be released without phosphorylation, modeled as a jump from the adhesion site to $z = 2$, with rate $R_{u,YAP}$. The value of $R_{u,YAP}$ is estimated based on the experimental findings for FAK and paxillin. The reported residence times for FAK and paxillin at focal adhesions are approximately 60 and 120 s, respectively (34). Additionally, the half-time recovery for FAK has been measured at 9.9 and 17 s (35,36). We therefore estimated the unbinding rates as $1/t\_immobile$, where $t\_immobile$ represents the average residence time of the protein in adhesion complexes in experiment, resulting in values ranging from $R_{u,YAP} = 0.01 - 0.1\ s^{-1}$. This is also in line with experimental findings that show the dissociation rates of various early adhesion proteins mostly fall between 0.02 and 0.2 s^{-1} (37). For the baseline model, we chose $R_{u,YAP} = 0.1\ s^{-1}$. In Figs. 3 and 4 we perform simulations with different unbinding rates that fall within the estimated range.

It has been shown that exposed binding sites on talin can prompt rapid vinculin binding and activation (38). Taking this as a representative case of adhesion-related factor binding and activation, in our model we assume that the phosphorylation rate $R_p = 200\ s^{-1} \gg R_{u,YAP}$; thus, the bound YAP gets phosphorylated with a higher chance than getting released without phosphorylation. Notably, YAP and pYAP within the adhesion do not experience diffusion, mimicking possible steric constraints within adhesions.

Release of pYAP from the adhesions

pYAP release, modeled as pYAP jump from adhesion sites to $z = 2$ in the z direction, occurs in two ways: 1) pYAP release from the adhesion characterized by the rate $R_{u,pYAP}$, and 2) for cases where a lifetime is assigned to adhesions (results in Fig. 4), pYAP and YAP within adhesions are released when the adhesion disassembles. In the baseline simulation, the unbinding rates of YAP and pYAP are the same ($R_{u,pYAP} = R_{u,YAP} = 0.1\ s^{-1}$), but we explore the effect of different unbinding rates in Figs. 3 and 4. Our model does not account for subsequent pYAP re-binding to adhesions following their release (only YAP can bind).

Adhesion turnover

Adhesions form and disassemble at varying intervals within the cell. In our model, to prevent the synchronization of adhesion turnover times, a random number t_l between 0 and lifetime is assigned to each adhesion initially, which determines the time it takes ($lifetime - t_l$) for the adhesion to disassemble. Nascent adhesions generally emerge within a fairly narrow size range, usually less than 1 μm in diameter (27). These adhesions also assemble and turnover at comparable rates (26). We therefore assumed that, after the turnover of small adhesions, a new adhesion of the same size emerges at a different random location, with its corresponding t_l value set to 0. By making this

assumption, we also fix the area covered by the adhesions while not considering the early stages of the formation of nascent adhesions.

The lifetime of focal adhesions generally follows a gamma distribution (39). However, for small nascent adhesions, a detailed lifetime distribution has not been experimentally measured. Therefore, we chose to perform simulations using the same lifetime for all adhesions. Nascent adhesions typically disassemble within approximately 2 min (26,40). The disassembly process begins roughly 60 s after formation, and the average measured lifetime of nascent adhesions is 135 ± 30 s (26,40). Therefore, we conducted simulations with lifetime values of 60 and 135 s.

Pairwise distance between binding sites

To quantify the distribution of integrin adhesions, we calculate the average pairwise distance between adhesion binding sites (yellow beads in Fig. 1) using the following expression:

$$\textit{Average pairwise distance} = \frac{2}{m(m-1)} \sum_{i=1}^{m-1} \sum_{j=i+1}^{m} d_{ij},$$

where m is the number of binding sites and d_{ij} represents the Euclidean distance between binding sites i and j (measured between the centers of the yellow beads). In Fig. S5, a second-order polynomial is employed to fit the pYAP ratio plotted against the average pairwise distance.

RESULTS AND DISCUSSION

pYAP steady-state levels depend on the number of binding sites, integrin adhesion spatial distribution, and diffusion rate

Considering that the accumulation of pYAP Y357 (referred to as pYAP in this study) by numerous small adhesions, as recently reported (13), is a novel mechanism previously unexplored in the literature for YAP (in contrast to the well-studied signaling cascade through serine 127 phosphorylation), the goal of this study is to use a particle-based stochastic model to provide a theoretical foundation for the experimental observations and concrete suggestions for experimental validation.

To explore the impact of adhesion size and number on the pYAP levels at steady state, we conducted a series of simulations with varying adhesion sizes and adhesion numbers using the same reaction and diffusion rates (see parameter set in Table S1). In each simulation, all adhesions have the same size, and the simulation is repeated three times starting from new random positions of the adhesions. Results in Fig. 2 *a* show that a larger adhesion, with size 9 × 9 (equivalent to 3.24 μm^2), results in a higher pYAP level at steady state in comparison to a smaller adhesion with size 3 × 3 (equivalent to 0.36 μm^2). This can be explained by the greater number of adhesion binding sites available on the membrane for YAP interaction when a larger adhesion is present. Similarly, increasing the number of binding sites by increasing the number of adhesions results in higher pYAP levels (see Fig. 2 *a*). In Fig. S6, we show a linear relationship between the total area covered by small adhesions and pYAP ratio. This is consistent with the previous linear trend observed between FAK phosphorylation and number of clustered integrins (14).

Comparing the results in Fig. 2 *a* for nine smaller adhesions ($N = 9$, size 3 × 3) and one larger adhesion

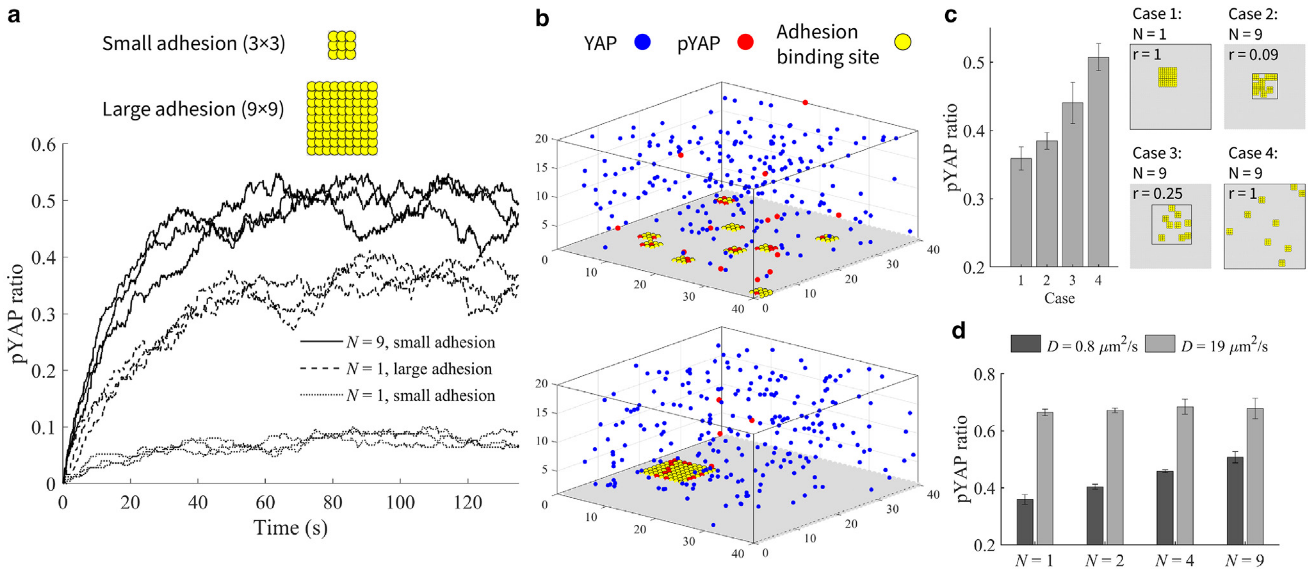

FIGURE 2 The effect of number of binding sites, adhesion spatial distribution, and diffusion rate on pYAP level. (*a*) Temporal evolution of pYAP ratio, defined as the count of pYAP divided by the total YAP count, for one and nine small adhesions ($N = 1{,}9$) of size 3 × 3 (0.36 μm^2) and one large adhesion ($N = 1$) of size 9 × 9 (3.24 μm^2) at $D = 0.8\ \mu m^2/s$ for $r = 1$. (*b*) Simulation box for two cases: nine small adhesions $N = 9$ and one large adhesion $N = 1$. (*c*) Comparing pYAP ratio for one large and nine small adhesions for cases where smaller adhesions are confined to be within $r = 1$ (case 1,4), $r = 0.09$ (case 2), and $r = 0.25$ (case 3) of the membrane area indicated by black squares. (*d*) pYAP ratio at steady state for different number of adhesions (N) that all cover the same total adhesion surface area on the plasma membrane at two different diffusion rates. The full parameter set can be found in Table S1. Error bars represent standard deviations calculated from three rounds of simulations. To determine the statistical significance between each pair of bars in (*c*) and (*d*) we report the p values in Table S4.

($N = 1$, size 9×9) shows that the number of binding sites (or the total adhesion area) is not the only factor influencing the pYAP level (see also snapshots of the simulation box for these two cases in Fig. 2 *b*). To explore what leads to a higher level of pYAP for smaller adhesions, we investigated scenarios where smaller 3×3-sized adhesions are confined to randomly chosen positions within $r = 0.25$ or 0.09 of the membrane area (Fig. 3 *c*). The outcomes show that positioning the smaller adhesions in closer proximity leads to a decrease in pYAP levels. Notably, distributing the small adhesions into 0.09 of the membrane area (12×12-sized area) leads to a pYAP level comparable with that of the single, larger 9×9-sized adhesion. To quantify the spatial distribution of adhesions, we calculated the average pairwise distance between adhesion binding sites. A higher average pairwise distance corresponds to a case where adhesions are distributed over a larger area (see "materials and methods" section). As demonstrated in Fig. S5, an increase in the average pairwise distance corresponds to an increase in the pYAP ratio. These results indicate that the spatial distribution of adhesions also affects the pYAP ratio. Distributing adhesions over a larger area increases the probability of YAP finding an adhesion site for binding while randomly diffusing in the simulation domain. In Fig. S7, we explore the impact of various YAP concentrations ($n_{YAP} = 150, 250, 400, \text{and } 800$) on pYAP levels and demonstrate that increasing the YAP concentration reduces the influence of adhesion spatial distribution on the pYAP level.

The impact of adhesion spatial distribution is anticipated to depend on the diffusion rate, as changes in diffusion rates directly affect how rapidly YAP can move within the box to locate an adhesion site. The formation of phase-separated condensates has also been shown to reduce YAP diffusion rate (41). Moreover, it is possible that high actin concentration and the presence of adaptor proteins around the adhesion sites could potentially slow down the diffusion of YAP, similar to the observed several-fold reduction in paxillin diffusion rate near adhesions compared to its cytoplasmic diffusion rate (32,33). Therefore, to explore the effect of diffusion, we performed our simulations for two different diffusion rates: $D = 0.8$ and $19\ \mu m^2/s$ (15,17,31). For simplicity, we consider the same diffusion rate for YAP in the entire simulation box except within the adhesions where YAP and pYAP are not allowed to diffuse. We also perform simulations for four cases with number of adhesions $N = 1, 2, 4, \text{and } 9$ that all occupy the same total adhesion surface area. In each case, all adhesions have the same size ($N = 1$ (total adhesion area is 3.24 μm^2), $N = 2$ (total adhesion area is $2 \times 1.62\ \mu m^2$), $N = 4$ (total adhesion area is $4 \times 0.81\ \mu m^2$), and $N = 9$ (total adhesion area is $9 \times 0.36\ \mu m^2$)), and the simulation is repeated three times starting from new random positions for the adhesions. Similar to Fig. 2 *c*, the results in Fig. 2 *d* show that, at the lower diffusion rate $D = 0.8\ \mu m^2/\ s$, reducing the size of the adhesions (hence increasing the number of adhesions and their spatial distribution) results in a higher pYAP level. As the diffusion increases to $D = 19\ \mu m^2/s$, the pYAP level increases due to the higher chance of YAP finding adhesion binding sites. At this higher diffusion rate, however, the impact of the spatial distribution of adhesions becomes less significant, resulting in a smaller contrast between pYAP levels (see Fig. 2 *d*). Increasing the diffusion rate increases the update frequency of YAP positions within the box, giving YAP a similar chance of being close to either the clumped-together or well-distributed adhesions across the plasma membrane. In summary, our simulation results show that the pYAP steady-state levels increase with increasing number of binding sites, spatial distribution of the adhesions across the membrane, and diffusion rate.

YAP binding rate has a significant effect on pYAP level at steady state

YAP is shown to be recruited to small focal complexes and phosphorylated at the Y357 residue, whereas adhesion maturation into focal adhesions dramatically reduces pYAP levels (13). Although not yet experimentally confirmed, a mechanistic interpretation proposes that, during the initial stages of adhesion maturation, YAP-binding sites within (nascent or small) adhesions might become accessible. These sites could become concealed as the adhesion matures and focal adhesions form, resulting in a decrease in pYAP levels. In our model, the amount of YAP recruited to the adhesions, thus becoming available to undergo phosphorylation, depends on two rates: the rate of YAP binding to the adhesions R_b and the rate of YAP unbinding from the adhesions without phosphorylation $R_{u,YAP}$ (see the "materials and methods" section for more details). We therefore conducted a series of simulations to explore the effect of these two rates on pYAP levels. We performed simulations for two sizes of adhesions, namely one large and nine small adhesions, similar to Fig. 2 *b*, which occupy the same total adhesion surface area. The findings in Fig. 3 *a* show that decreasing the binding rate R_b leads to a reduction in pYAP level, with a more pronounced effect observed for smaller adhesions ($N = 9$). However, increasing $R_{u,YAP}$ up to twofold ($R_{u,YAP} = 0.2\ s^{-1}$) (37) has negligible impact on pYAP level. This can be explained by considering that, in our model, the rate of phosphorylation within the adhesions R_p is significantly higher than the unbinding rate of YAP. As a result, the influence of increasing $R_{u,YAP}$ on pYAP levels is comparatively limited.

In Fig. 3 *a*, we see that, when considering a higher binding rate to small adhesions compared to larger adhesions, the previously discussed differences in pYAP levels are amplified. This is evident when comparing the pYAP ratio for $N = 9, R_b = 50\ s^{-1}$ (small adhesions) to the pYAP ratio for $N = 1,\ R_b = 10, 50\ s^{-1}$ (large adhesion) in Fig. 3

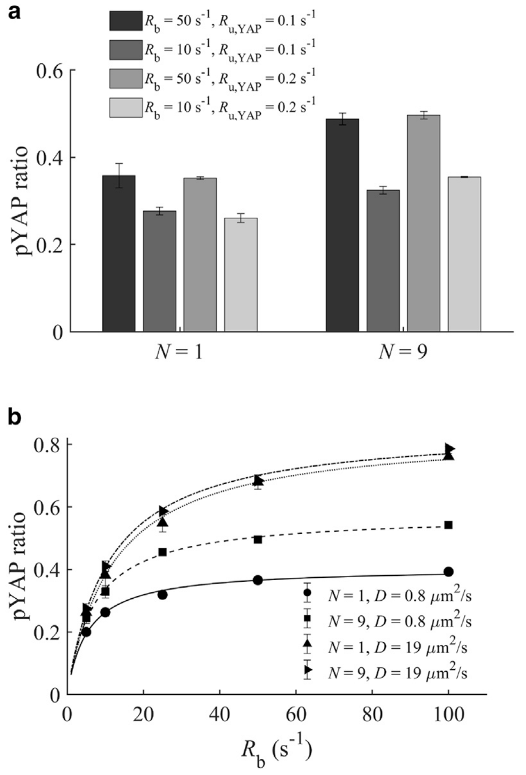

FIGURE 3 The effect of YAP-adhesion binding and unbinding rates on pYAP level. (*a*) Comparing the effect of YAP binding rate (R_b = 10, 50 s^{-1}) and unbinding rate ($R_{u,YAP}$ = 0.1, 0.2 s^{-1}) for two adhesion sizes (N = 1 large and N = 9 small) using D = 0.8 μm^2/s. (*b*) pYAP ratio for two adhesion sizes (N = 1 large and N = 9 small) plotted against the pYAP binding rate (R_b) at D = 0.8, 19 μm^2/s and $R_{u,YAP}$ = 0.1 s^{-1}. For the full parameter set, see Table S2. Error bars represent standard deviations calculated from three rounds of simulations.

a. Furthermore, Fig. 3 *a* demonstrates that the influence of the spatial distribution of adhesions, as depicted in Fig. 2 *c* and *d*, becomes less prominent as binding rates decrease. For example, compare the differences between the cases with R_b = 10 s^{-1} and the differences between the cases with R_b = 50 s^{-1} for N = 1 and N = 9. These findings highlight the critical impact of the YAP binding rate on the pYAP level.

To further explore the impact of the binding rate, in Fig. 3 *b* we show results for the pYAP levels across a range of binding rates spanning from R_b = 5 to 100 s^{-1}. These results are then fitted with the following Hill-type function:

$$pYAP\ ratio = \frac{k1R_b}{k2 + R_b},$$

where $k1$ and $k2$ are fitting parameters (see Table S5 in the Supporting Material). Notably, at lower binding rates, small and large adhesions display similar levels of pYAP. However, with increasing binding rates, the differences in pYAP levels between small and large adhesions become more pronounced for diffusion rate D = 0.8 μm^2/s. This observation implies that, when assuming identical (low) diffusion rates around both small and large adhesions, as well as the same binding rate to these adhesions, achieving a significant divergence in pYAP levels requires a binding rate $R_b > 20$ s^{-1} with the adhesions.

As depicted in Fig. 2 *d*, at a higher diffusion rate D = 19 μm^2/*s*, with the same binding rate for small and large adhesions (N = 1, 9), the effect of adhesion size is negligible. However, considering a higher binding rate for small adhesions compared to the larger ones leads to higher pYAP levels for smaller adhesions even at a higher diffusion rate (D = 19 μm^2/s); see, for example, the pYAP ratio at R_b = 50 s^{-1} and R_b = 10 s^{-1} for D = 19 μm^2/s (Fig. 3 *b*). In summary, the YAP level increases with increasing binding rate, which can be further amplified if the binding rate is dependent on the adhesion size (i.e., higher for small focal complexes).

The effect of lifetime on pYAP level depends on the mechanism of pYAP release from the adhesions

Nascent adhesions have been observed to initiate disassembly after approximately 60 s, with an average reported lifetime of around 135 ± 30 s (26,40). In the context of YAP phosphorylation within adhesions, it is reasonable to assume that both YAP and pYAP are released as adhesions disassemble. We therefore explore the impact of assigning a lifetime to adhesions on pYAP levels (see the "materials and methods" section for the modeling details). We performed this analysis on smaller adhesions since focal adhesions typically have a lifetime of tens of minutes (42), and therefore larger adhesions do not disassemble within the time frame of our simulations. In Fig. 4, we present our findings for N = 9 small adhesions, covering three cases: no adhesion turnover and adhesion turnover with lifetimes of 60 and 135 s. The figure reports total pYAP ratios similar to Figs. 2 and 3, as well as pYAP ratio both within and outside adhesions. Given that assigning lifetimes to adhesions influences the manner in which pYAP is released from adhesions, we also show results across a range of pYAP unbinding rates ($R_{u,pYAP}$ = 0.001 − 0.2 s^{-1}), which is the rate of pYAP release from the adhesion independent of adhesion disassembly. This helps us explore the interplay between these two factors ($R_{u,pYAP}$ and adhesion lifetime) and their impact on pYAP levels. It is important to note that, when $R_{u,pYAP}$ is exceedingly low (approaching zero), pYAP release predominantly occurs upon adhesion turnover.

In Fig. 4 (black curves), the results show that, as $R_{u,pYAP}$ increases, the total pYAP ratio and pYAP outside the adhesions increase, whereas the pYAP within the adhesions decreases across all cases. pYAP ratios inside/outside the adhesions are the count of pYAP inside/outside of the adhesions divided by the total count of YAP. At $R_{u,pYAP}$ = 0.1 s^{-1}, the rate used in Figs. 2 and 3, the effect of adhesion lifetime is minimal,

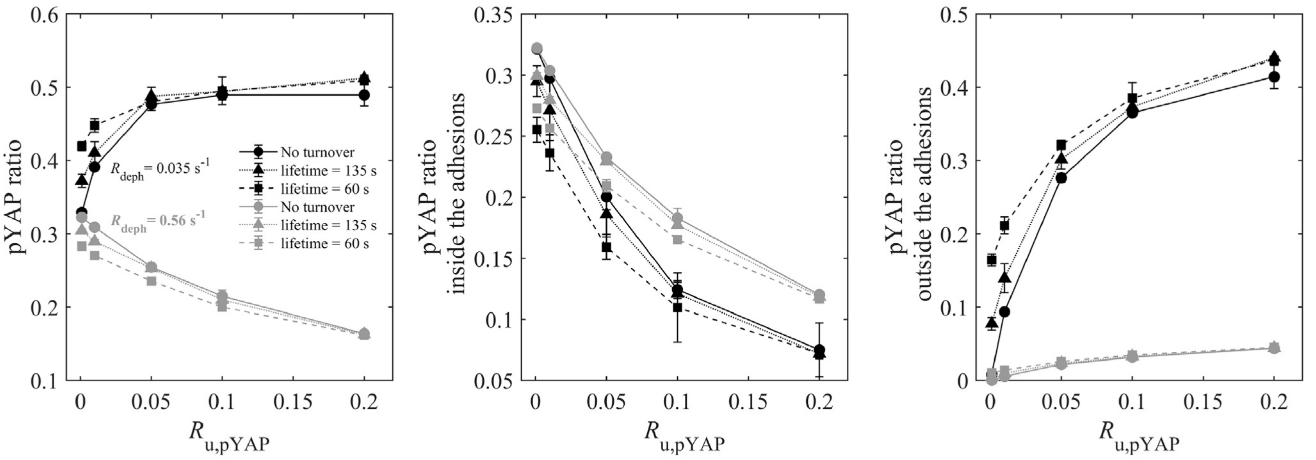

FIGURE 4 The effect of lifetime and pYAP unbinding rate from adhesions on pYAP level. The total pYAP ratio, pYAP ratio within the adhesions (calculated as pYAP inside adhesion/total YAP), and pYAP ratio outside the adhesions (calculated as pYAP outside adhesion/total YAP) for nine small adhesions $N = 9$ for three cases: no adhesion turnover, and lifetimes of 60 and 135 s for two the different dephosphorylation rates $R_{deph} = 0.035$ s^{-1} (similar to Figs. 2 and 3) (*black*), and $R_{deph} = 0.56$ s^{-1} (*gray*). For the full parameter set, see Table S3. Error bars represent standard deviations calculated from three rounds of simulations.

and the three cases predict similar pYAP ratios (total, within, and outside the adhesions); see also the temporal evolution of pYAP in Fig. S8. However, for lower $R_{u,pYAP}$ values, especially those close to zero (<0.01 s^{-1}), adhesion disassembly increases the total pYAP ratio and pYAP ratio outside the adhesions but concurrently reduces pYAP within the adhesions (see Fig. S9 black curves for zoomed-in panels of Fig. 4). In understanding these findings, it is crucial to recognize the interplay of two contrasting mechanisms: 1) with no adhesion turnover, an increasing number of binding sites within the adhesions become occupied, reducing the overall available binding sites for YAP. In contrast, as adhesions disassemble and new ones emerge, new binding sites become available, and a lower lifetime increases the frequency of this process. 2) pYAP within the adhesions does not undergo dephosphorylation in the stochastic model; consequently, a higher pYAP concentration within the adhesions protects against dephosphorylation. In Fig. 4 (black curves), the dominance of the first scenario becomes evident, resulting in a higher total pYAP at low $R_{u,pYAP}$ values when adhesion disassembly is introduced. Increasing the lifetime reduces the frequency of pYAP release upon adhesion turnover and the emergence of new binding sites, as depicted by the curve behavior for a lifetime of 135 s between the other two scenarios; see in Fig. S10 the temporal evolution of total pYAP and pYAP within and outside the adhesions at $R_{u,pYAP} = 0.001$ s^{-1}.

We also conducted tests using a higher (cytosolic) dephosphorylation rate $R_{deph} = 0.56$ s^{-1} (Fig. 4, gray curves), which was previously used in a computational model for YAP mechanotransduction (15). A higher dephosphorylation leads to a reduction in pYAP levels as expected (Fig. 4, left panel). Notably, as depicted in Fig. 4 (gray curves), with a higher dephosphorylation rate, there is a reversal in the trend seen previously regarding the total pYAP ratio as $R_{u,pYAP}$ increases. This behavior can be explained by considering that, with a higher dephosphorylation rate, an increased release of pYAP significantly amplifies the probability of their dephosphorylation. At lower values of the release rate $R_{u,pYAP}$, there is a higher accumulation of pYAP within the adhesions, which enhances the pYAP ratio by protecting them from dephosphorylation. At $R_{u,pYAP}$ values close to zero, where the influence of the adhesion lifetime is more pronounced, and release primarily occurs upon adhesion turnover, the less frequent release of pYAP results in a higher pYAP ratio. This is because, after release, there is a higher likelihood of subsequent dephosphorylation.

In summary, the impact of adhesion lifetime on the pYAP level is most pronounced when the $R_{u,pYAP}$ is small (<0.01 s^{-1}). With a lower dephosphorylation rate in the cytoplasmic region, high adhesion turnover leads to an increase in the pYAP ratio, whereas, with a higher dephosphorylation rate, high adhesion turnover decreases the pYAP ratio.

Conclusions

Various mechanical and biochemical cues have been demonstrated to initiate signaling cascades, resulting in YAP translocation to the nucleus where it regulates gene expression by binding to transcription factors. Our recent research has uncovered a novel role of YAP in activating apoptosis, which begins with the direct recruitment of YAP to small adhesions, followed by YAP phosphorylation at the Y357 residue—a mechanism not observed in mature focal adhesions (13). However, it is unclear why Y357 pYAP levels are higher in small adhesions than in mature

focal adhesions. We have developed a computational framework to explore potential explanations for the observed behavior in the experiments (see Fig. 5).

We show that distributing the adhesions across the plasma membrane results in higher pYAP levels. This observation highlights the efficiency of smaller adhesions in generating higher pYAP levels than larger focal adhesions. This observation is particularly prominent at a lower diffusion rate of YAP. Additionally, we illustrate that increasing the diffusion rate of YAP leads to an overall increase in pYAP levels. Assuming a reduction in diffusion rate around focal adhesions (32), our findings indicate that a lower diffusion rate of YAP around larger focal adhesions—compared to smaller adhesions—could potentially contribute to the observed lower pYAP levels in focal adhesions (13). Future work should focus on experimentally exploring this computational prediction using, e.g., fluorescence recovery after photobleaching experiments at very short timescales (2–3 s overall) to measure the diffusion rate in the juxtamembrane cytoplasmic regions in small vs. mature adhesions (32).

Our findings also highlight the significant effect of the YAP binding rate to adhesions (R_b) on pYAP levels. As the binding rate increases, the difference in pYAP levels between small and large adhesions becomes more pronounced. Assigning a higher YAP binding rate to small adhesions (lower binding rate to larger adhesions) can lead to increased pYAP levels for smaller adhesions even at a higher diffusion rate. This suggests that the experimentally observed differences in the recruitment of YAP to small and large adhesions could potentially come from the higher YAP binding rate to small adhesions. Testing this experimentally can also be done in principle using fluorescence recovery after photobleaching; however, such measurements will be more challenging with respect to small adhesions as this will require measurements on timescales of a few tens of seconds (32), which is in the same range of small adhesions' lifetimes. In addition, this finding may suggest inherent differences in the types of binding sites that are available for YAP in small versus large adhesions, although further work is needed to confirm this. Nevertheless, although binding sites and affinity may be different, our results also highlight that different YAP levels can be predicted, even with the same binding rates to small and large adhesions, when varying the YAP diffusion rate and adhesion spatial distribution.

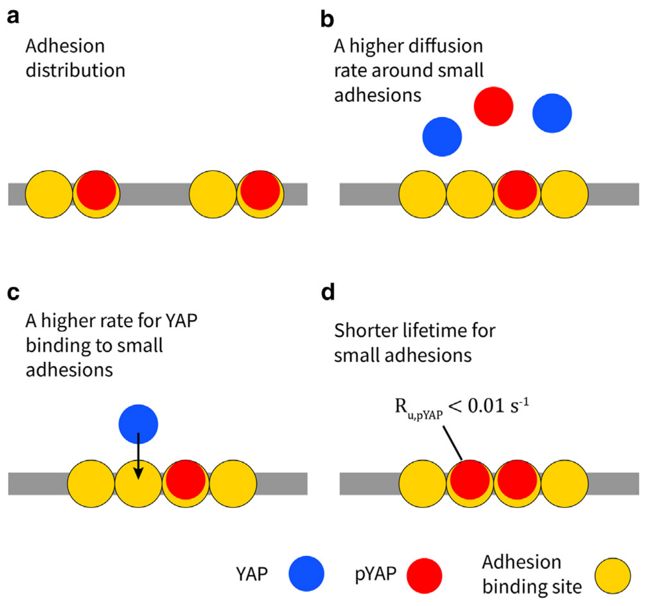

FIGURE 5 Summary of different factors that result in higher Y357 pYAP in small adhesions than in mature focal adhesions. (*a*) Distributing the adhesion across the plasma membrane, (*b*) a higher diffusion rate around small adhesions, and (*c*) a higher binding rate to small adhesions all result in a higher pYAP level for small adhesions. (*d*) For small adhesions, at a low $R_{u,pYAP}$ ($< 0.01\ s^{-1}$), a shorter lifetime also increases the pYAP level.

We further delve into the mechanism behind pYAP release from small adhesions. We show that at markedly lower $R_{u,pYAP}$ values ($< 0.01\ s^{-1}$) and under a lower (cytoplasmic) dephosphorylation rate, incorporating pYAP release upon adhesion turnover leads to an increase in pYAP levels. In this case, reducing the adhesion lifetime increases pYAP levels for small adhesions. It is important to note that this observation assumes the presence of new adhesions with unoccupied binding sites after turnover. The marked dependence on the dephosphorylation rate makes this parameter of high importance for the downstream transmission of the signals, especially when considering that the pYAP molecules need to translocate into the nucleus to activate the apoptotic response (12). Experimentally, testing the involvement of YAP dephosphorylation on the overall efficiency of the mechanotransduction process will require identifying the phosphate(s) involved and testing the cellular response upon their knockout/mutagenesis.

It is important to mention that our investigation did not explore the dynamics of adhesion growth. Combining the current model with earlier models of integrin clustering (14,43–46) could provide valuable insights into the regulatory mechanisms of pYAP initiated from numerous small adhesions, including the effect of matrix stiffness. Future modeling efforts could also include the lifetime distribution of smaller nascent adhesions as experimental data become available. Another limitation of our model is the impact of excluded volume repulsion between YAP proteins; i.e., only a single YAP can occupy a lattice site. We believe that the main impact of this repulsion assumption is on the YAP binding to the adhesion, representing a limited number of binding sites. It has been shown that phosphorylated forms of FAK and paxillin localize within specific sub-regions of focal adhesions (33,47). However, due to a lack of similar detailed measurements for (p)YAP, our model simplifies the binding-site distribution by considering adhesions with square arrangements and assigning a uniform probability of binding across these areas, similar to (14). Such additional complexities could readily be

incorporated once quantitative information on the amount and distribution of YAP-binding sites becomes available. Moreover, due to the absence of data on the binding and unbinding rates of YAP and pYAP to small adhesions, we were unable to quantitatively determine which contributing factor (as summarized in Fig. 5) plays a more significant role in the accumulation of pYAP by small adhesions. Nonetheless, our qualitative analysis offers new insights into the interplay between various factors and their collective influence on overall pYAP levels.

In summary, the results of this study contribute to an improved understanding of YAP mechanotransduction mechanisms as our findings highlight the complex interplay of several factors—YAP diffusion rate, adhesion size, spatial distribution and lifetime, and pYAP (un)binding rates to the adhesions—in influencing pYAP levels. We showed that the release mechanism (release due to the pYAP unbinding rate versus release due to adhesion disassembly) together with the dephosphorylation rate determines whether the adhesion turnover rate affects YAP phosphorylation level. When the unbinding rate is low and the main mechanism of pYAP release is adhesion disassembly, a higher turnover rate (shorter lifetime) can either increase or decrease the pYAP level depending on the rate of dephosphorylation outside the adhesions. These dynamics constitute an emergent phenomenon where the collective effects of these variables influence the pYAP ratio.

ACKNOWLEDGMENTS

We thank Iga Skorupska for independently performing a reproducibility check of the model simulations.

This work was financially supported by the Gravitation Program “Materials Driven Regeneration”, funded by the Netherlands Organisation for Scientific Research (024.003.013). H.W. acknowledges support by the Rappaport Family Foundation, the John Templeton Foundation (grant no. 62568; the opinions expressed in this publication are those of the authors and do not necessarily reflect the views of the John Templeton Foundation), and the German Research Foundation (DFG; grant no. GZ PO 1725/13-1 AOBJ 697448).

AUTHOR CONTRIBUTIONS

H.J., L.S., H.W., and A.C. designed research. H.J. performed and analyzed computational modeling. L.S. performed and analyzed experiments. H.J., L.S., H.W., and A.C. wrote the paper.

DECLARATION OF INTERESTS

The authors declare no competing interests.

SUPPORTING MATERIAL

Supporting material can be found online at https://doi.org/10.1016/j.bpj.2024.09.002.

REFERENCES

1. Misra, J. R., and K. D. Irvine. 2018. The hippo signaling network and its biological functions. *In* Annual Review of Genetics Annual Reviews Inc, pp. 65–87.
2. Dupont, S., L. Morsut, …, S. Piccolo. 2011. Role of YAP/TAZ in mechanotransduction. *Nature.* 474:179–183.
3. Heng, B. C., X. Zhang, …, X. Deng. 2021. An overview of signaling pathways regulating YAP/TAZ activity. *Cell. Mol. Life Sci.* 78:497–512.
4. Dasgupta, I., and D. McCollum. 2019. Control of cellular responses to mechanical cues through YAP/TAZ regulation. *J. Biol. Chem.* 294:17693–17706.
5. Elosegui-Artola, A., I. Andreu, …, P. Roca-Cusachs. 2017. Force Triggers YAP Nuclear Entry by Regulating Transport across Nuclear Pores. *Cell.* 171:1397–1410.e14.
6. Koushki, N., A. Ghagre, …, A. J. Ehrlicher. 2023. Nuclear compression regulates YAP spatiotemporal fluctuations in living cells. *Proc. Natl. Acad. Sci. USA.* 120:e2301285120.
7. Ghagre, A., A. Delarue, A. Ehrlicher…, 2024. Nuclear curvature determines Yes-Associated Protein nuclear localization and differentiation of mesenchymal stem cells. *Biophys. J.* 123:1222–1239.
8. Piccolo, S., S. Dupont, and M. Cordenonsi. 2014. The biology of YAP/TAZ: hippo signaling and beyond. *Physiol. Rev.* 94:1287–1312.
9. Varelas, X. 2014. The hippo pathway effectors TAZ and YAP in development, homeostasis and disease. *Development (Camb.).* 141:1614–1626.
10. Lin, K. C., H. W. Park, and K. L. Guan. 2017. Regulation of the Hippo Pathway Transcription Factor TEAD. *Trends Biochem. Sci.* 42:862–872.
11. Calvo, F., N. Ege, …, E. Sahai. 2013. Mechanotransduction and YAP-dependent matrix remodelling is required for the generation and maintenance of cancer-associated fibroblasts. *Nat. Cell Biol.* 15:637–646.
12. Levy, D., Y. Adamovich, …, Y. Shaul. 2008. Yap1 Phosphorylation by c-Abl Is a Critical Step in Selective Activation of Proapoptotic Genes in Response to DNA Damage. *Mol. Cell.* 29:350–361.
13. Shi, L., E. Nadjar-Boger, …, H. Wolfenson. 2024. YAP mediates apoptosis through failed integrin adhesion reinforcement. *Cell Rep.* 43:113811.
14. Cheng, B., W. Wan, …, M. Lin. 2020. Nanoscale integrin cluster dynamics controls cellular mechanosensing via FAKY397 phosphorylation. *Sci. Adv.* 6:eaax1909.
15. Scott, K. E., S. I. Fraley, and P. Rangamani. 2021. A spatial model of YAP/TAZ signaling reveals how stiffness, dimensionality, and shape contribute to emergent outcomes. *Proc. Natl. Acad. Sci. USA.* 118:e2021571118.
16. Eroumé, K. S., R. Cavill, …, A. Carlier. 2021. Exploring the influence of cytosolic and membrane FAK activation on YAP/TAZ nuclear translocation. *Biophys. J.* 120:4360–4377.
17. Wehling, L., L. Keegan, …, K. Breuhahn. 2022. Spatial modeling reveals nuclear phosphorylation and subcellular shuttling of YAP upon drug-induced liver injury. *Elife.* 11:e78540.
18. Elosegui-Artola, A., X. Trepat, and P. Roca-Cusachs. 2018. Control of mechanotransduction by molecular clutch dynamics. *Trends Cell Biol.* 28:356–367.
19. Sun, M., F. Spill, and M. H. Zaman. 2016. A computational model of YAP/TAZ mechanosensing. *Biophys. J.* 110:2540–2550.
20. Jafarinia, H., A. Khalilimeybodi, …, A. Carlier. 2024. Insights gained from computational modeling of YAP/TAZ signaling for cellular mechanotransduction. *npj Syst. Biol. Appl.* 10:90.
21. Cheng, B., M. Li, …, F. Xu. 2023. Predicting YAP/TAZ nuclear translocation in response to ECM mechanosensing. *Biophys. J.* 122:43–53.
22. Elf, J., and M. Ehrenberg. 2004. Spontaneous separation of bi-stable biochemical systems into spatial domains of opposite phases. *Syst. Biol.* 1:230–236.

23. Gillespie, D. T. 1976. A general method for numerically simulating the stochastic time evolution of coupled chemical reactions. *J. Comput. Phys.* 22:403–434.

24. Henning Stumpf, B., A. Ambriović-Ristov, …, A.-S. Smith. 2020. Recent advances and prospects in the research of nascent adhesions. *Front. Physiol.* 11:574371.

25. Sun, Z., A. Lambacher, and R. Fässler. 2014. Nascent adhesions: from fluctuations to a hierarchical organization. *Curr. Biol.* 24:R801–R803.

26. Choi, C. K., M. Vicente-Manzanares, …, A. R. Horwitz. 2008. Actin and α-actinin orchestrate the assembly and maturation of nascent adhesions in a myosin II motor-independent manner. *Nat. Cell Biol.* 10:1039–1050.

27. Wolfenson, H., I. Lavelin, and B. Geiger. 2013. Dynamic regulation of the structure and functions of integrin adhesions. *Dev. Cell.* 24:447–458.

28. Brami-Cherrier, K., N. Gervasi, …, T. Bouceba. 2014. FAK dimerization controls its kinase-dependent functions at focal adhesions. *EMBO J.* 33:356–370.

29. Xue, X., X. Tian, …, X. Zhang. 2022. YAP ISGylation increases its stability and promotes its positive regulation on PPP by stimulating 6PGL transcription. *Cell Death Dis.* 8:59.

30. Kumar, S., A. Das, and S. Sen. 2014. Extracellular matrix density promotes EMT by weakening cell–cell adhesions. *Mol. Biosyst.* 10:838–850.

31. Ege, N., A. M. Dowbaj, …, E. Sahai. 2018. Quantitative Analysis Reveals that Actin and Src-Family Kinases Regulate Nuclear YAP1 and Its Export. *Cell Syst.* 6:692–708.e13.

32. Wolfenson, H., A. Lubelski, …, B. Geiger. 2009. A role for the juxtamembrane cytoplasm in the molecular dynamics of focal adhesions. *PLoS One.* 4:e4304.

33. Digman, M. A., C. M. Brown, …, E. Gratton. 2008. Paxillin dynamics measured during adhesion assembly and disassembly by correlation spectroscopy. *Biophys. J.* 94:2819–2831.

34. Le Dévédec, S. E., B. Geverts, …, B. van De Water. 2012. The residence time of focal adhesion kinase (FAK) and paxillin at focal adhesions in renal epithelial cells is determined by adhesion size, strength and life cycle status. *J. Cell Sci.* 125:4498–4506.

35. Stutchbury, B., P. Atherton, …, C. Ballestrem. 2017. Distinct focal adhesion protein modules control different aspects of mechanotransduction. *J. Cell Sci.* 130:1612–1624.

36. Giannone, G., P. Rondé, …, K. Takeda. 2004. Calcium rises locally trigger focal adhesion disassembly and enhance residency of focal adhesion kinase at focal adhesions. *J. Biol. Chem.* 279:28715–28723.

37. Lavelin, I., H. Wolfenson, …, B. Geiger. 2013. Differential effect of actomyosin relaxation on the dynamic properties of focal adhesion proteins. *PLoS One.* 8:e73549.

38. Wang, Y., M. Yao, …, J. Yan. 2021. Force-dependent interactions between talin and full-length vinculin. *J. Am. Chem. Soc.* 143:14726–14737.

39. Rosen, M. E., and J. C. Dallon. 2022. A mathematical analysis of focal adhesion lifetimes and their effect on cell motility. *Biophys. J.* 121:1070–1080.

40. Changede, R., X. Xu, …, M. P. Sheetz. 2015. Nascent integrin adhesions form on all matrix rigidities after integrin activation. *Dev. Cell.* 35:614–621.

41. Hao, S., H. Fuehrer, …, D. Cai. 2022. YAP condensates are highly organized hubs for YAP/TEAD transcription. Preprint at bioRxiv. https://doi.org/10.1101/2022.10.24.513621.

42. Stehbens, S. J., and T. Wittmann. 2014. Analysis of focal adhesion turnover: a quantitative live-cell imaging example. *In* Methods in Cell Biology Elsevier, pp. 335–346.

43. Bidone, T. C., A. V. Skeeters, …, G. A. Voth. 2019. Multiscale model of integrin adhesion assembly. *PLoS Comput. Biol.* 15:e1007077.

44. Honasoge, K. S., Z. Karagöz, …, A. Carlier. 2023. Force-dependent focal adhesion assembly and disassembly: A computational study. *PLoS Comput. Biol.* 19:e1011500.

45. Comisar, W. A., D. J. Mooney, and J. J. Linderman. 2011. Integrin organization: linking adhesion ligand nanopatterns with altered cell responses. *J. Theor. Biol.* 274:120–130.

46. Brinkerhoff, C. J., and J. J. Linderman. 2005. Integrin dimerization and ligand organization: key components in integrin clustering for cell adhesion. *Tissue Eng.* 11:865–876.

47. Bachmann, M., A. Skripka, …, M. Bastmeyer. 2022. Phosphorylated paxillin and phosphorylated FAK constitute subregions within focal adhesions. *J. Cell Sci.* 135:jcs258764.

Biophysical Journal, Volume *123*

Supplemental information

YAP phosphorylation within integrin adhesions: Insights from a computational model

Hamidreza Jafarinia, Lidan Shi, Haguy Wolfenson, and Aurélie Carlier

Supplemental information

Supplemental figures

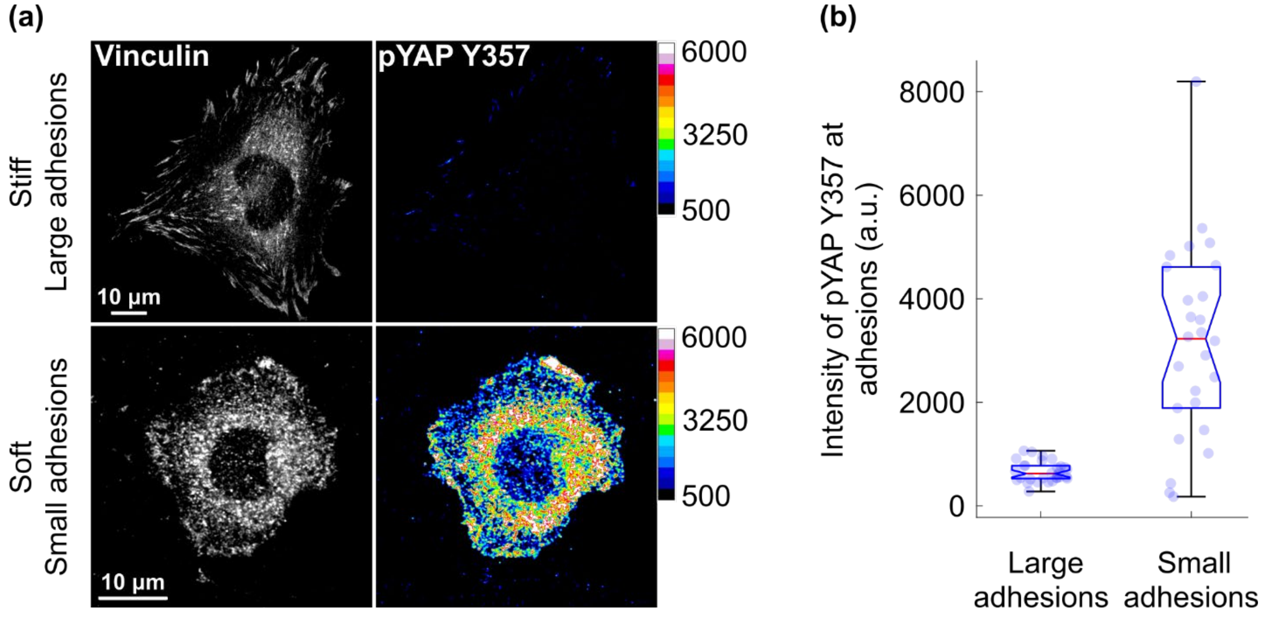

Figure S1: Experimental results for the intensity of pYAP at adhesions

(a) Representative confocal microscopy images for MEF cells that were seeded on fibronectin-coated silicon gels for 3 hours and immunostained for vinculin (grayscale, left) and pYAP Y357 (color coded for intensity, right) on stiff (top) and soft (bottom) substrates. Vinculin is a marker for adhesions. (b) Quantification of pYAP Y357 intensity within small and large adhesions in cells (N = 30 cells in each case) from the experiment shown in panel (a). Red lines in the boxplots are the median values; the bottom and top edges of the blue box represent the 25th and 75th percentiles, respectively; and the black top and bottom represent the minimal and maximal values that are not outliers, respectively. Data points are represented as blue scattered dots. Each data point represents the average value of a cell.

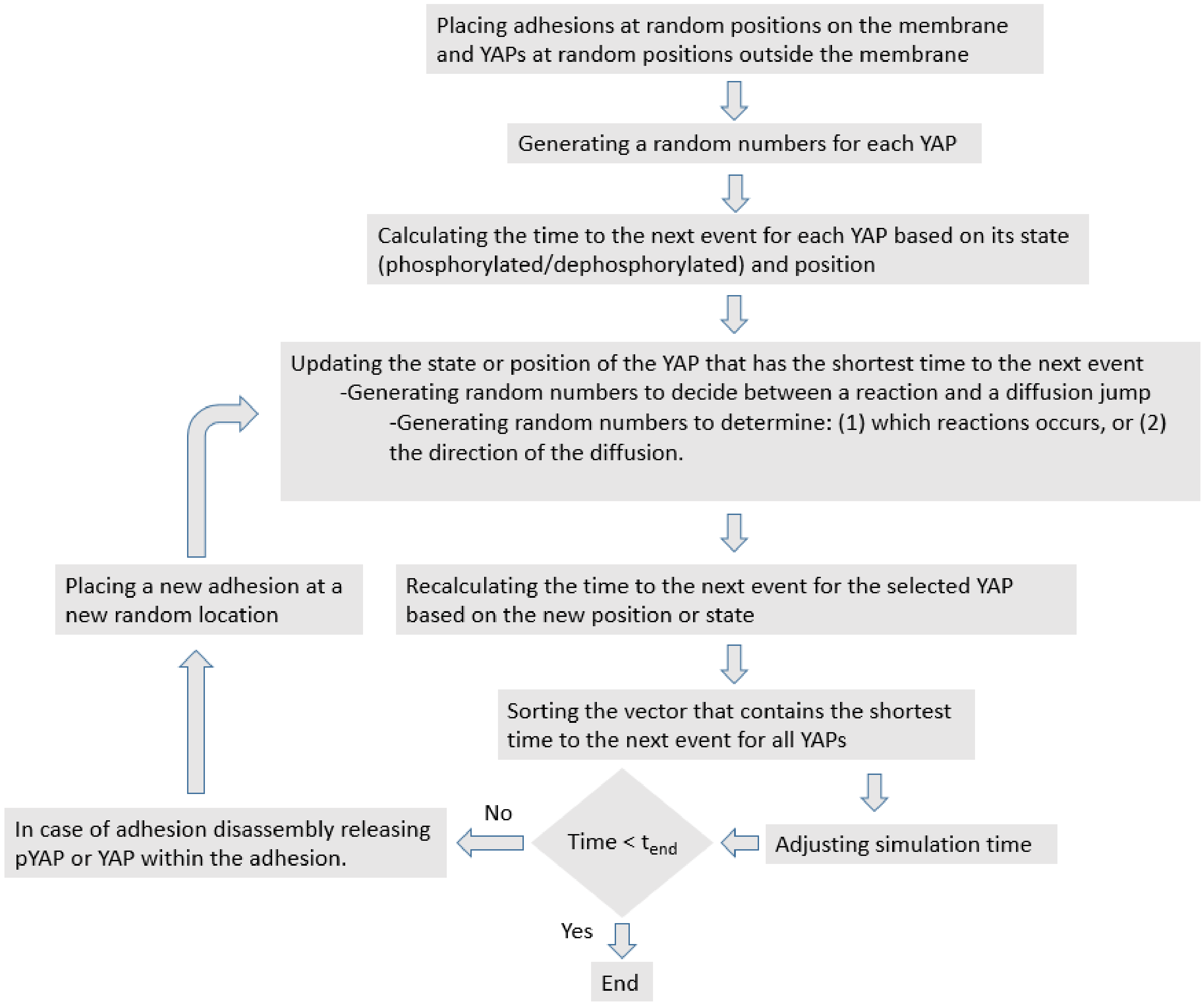

Figure S2: Simulation algorithm

See the methods section for more details. The value of t_{end} is larger than the time it takes for pYAP to reach steady state condition.

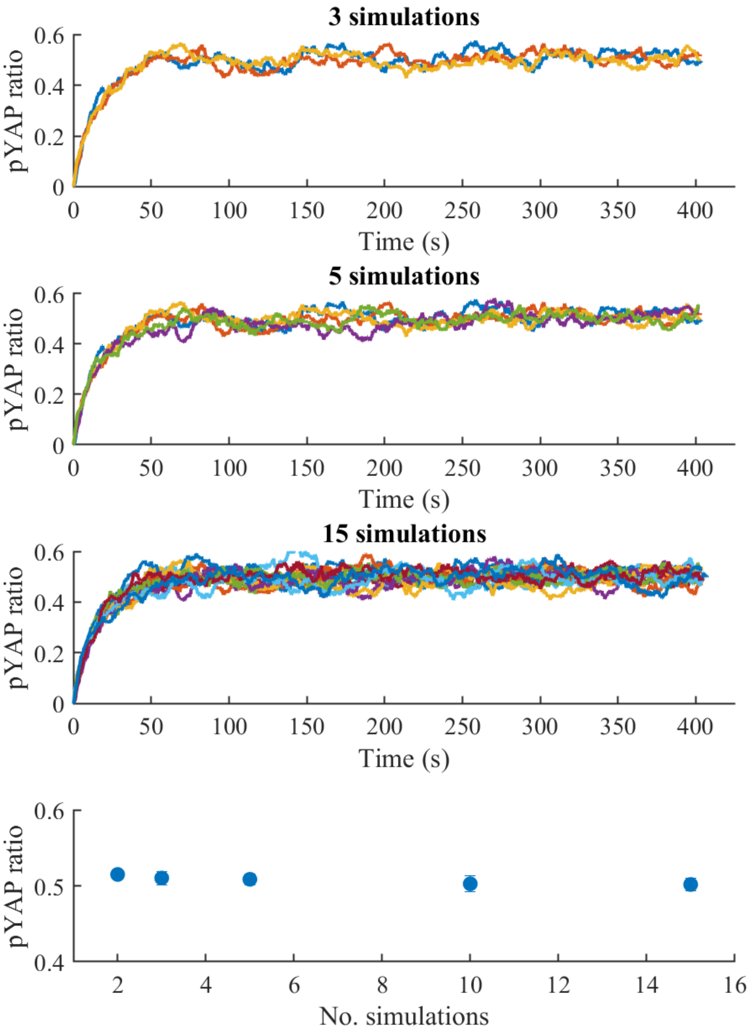

Figure S3: The average pYAP level calculated for different number of replicates

(Top panels) Temporal evolution of pYAP ratio for a set of 5, 10, and 15 simulations (Bottom panel) average pYAP ratio plotted against the number of replicates for nine small adhesions ($N = 9$), $D = 0.8\ \mu m^2/s$. For the full parameter set see Table S1.

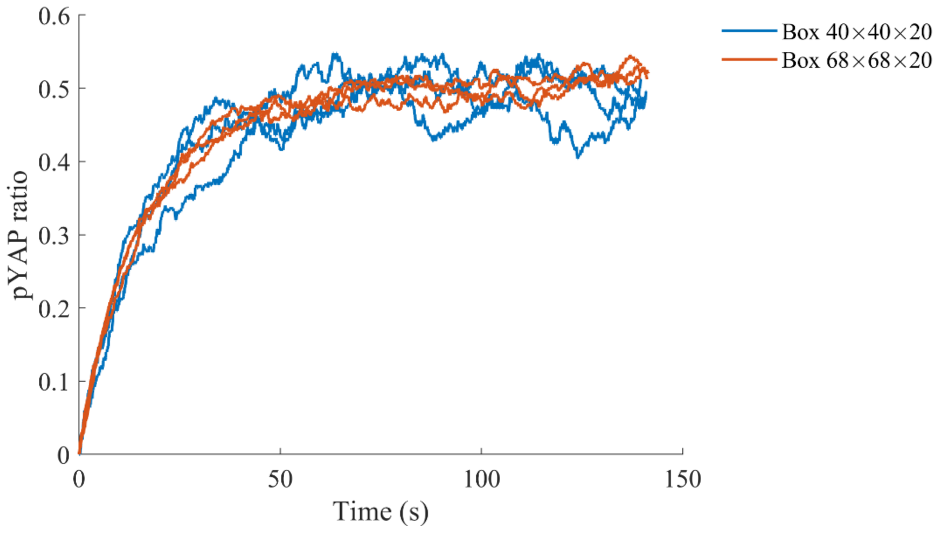

Figure S4: Comparing pYAP ratio for simulations in a larger box

Comparing pYAP ratio for simulations conducted in a larger box, 68x68x20, with 26 small adhesions (each size 3x3) and 722 YAP, with our previous simulations in a box of 40x40x20, with 9 small adhesions (each size 3x3) and 250 YAP. In both simulations the same percentage of the membrane is covered by adhesions. Additionally, the YAP concentration and the number of YAP per adhesion site are the same in both simulations. Note that the box size is not extended in z-direction to avoid changing the ratio of YAP count per adhesion site while keeping the YAP concentration constant. In these simulations $D = 0.8\,\mu m^2/s$ (for other parameters see table S1).

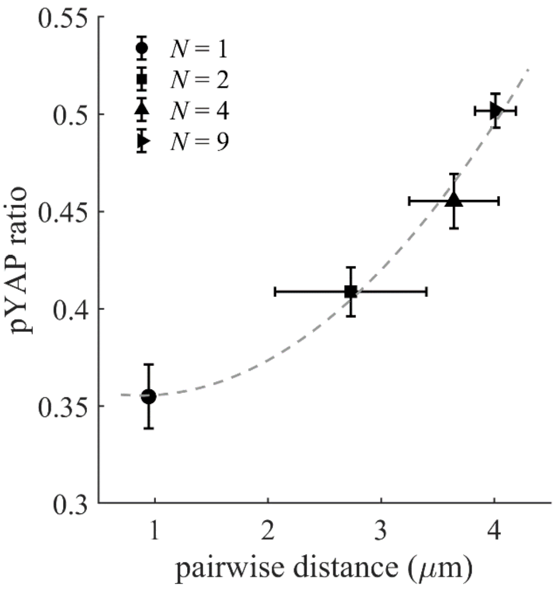

Figure S5: pYAP ratio plotted against the average pairwise distance of integrin adhesions binding sites for $N = 1, 2, 4, 9$ adhesions that all occupy the same surface area on the membrane

A second-order polynomial ($0.0143x^2 - 0.0250x + 0.3664$) is used to fit the dataset. The vertical and horizontal error bars are standard deviations calculated from 15 simulations each with a different random positions of adhesions. For a single adhesion $N = 1$, the pairwise distance remains the same across all simulations.

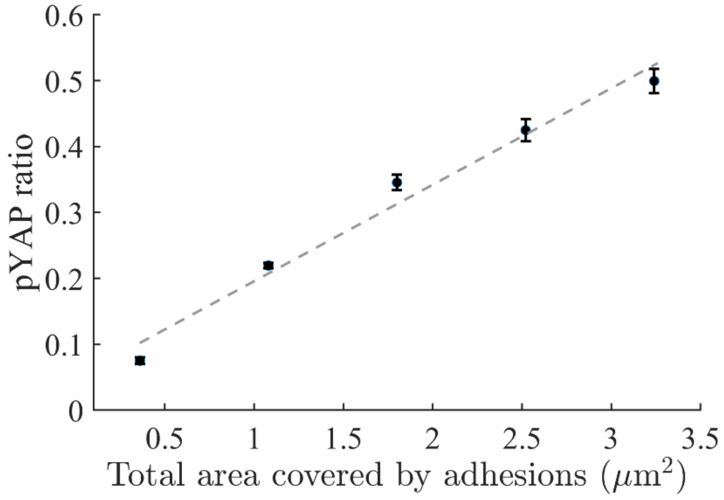

Figure S6: pYAP ratio plotted against the total area covered by adhesions

Data points correspond to 1, 3, 5, 7, and 9 small adhesions (each with a size of 3x3 or 0.36 μm^2). A linear fit ($0.1464x + 0.0494$) is used to fit the dataset. The error bars are standard deviations calculated from three simulations each with a different random positions of adhesions. The diffusion rate is set to $D = 0.8\ \mu m^2/s$ (for other parameters see table S1).

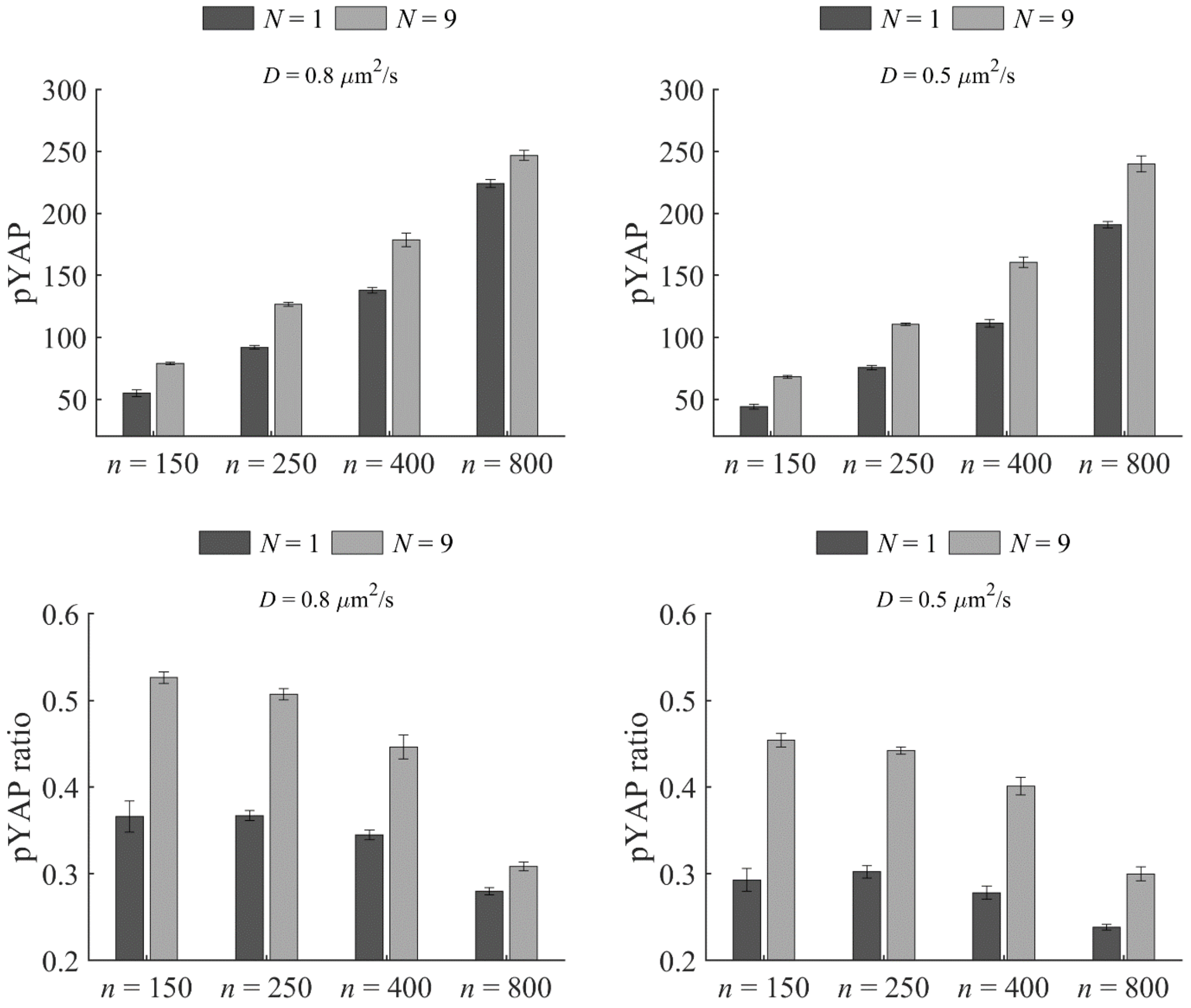

Figure S7: pYAP level plotted for different concentrations of total YAP

(Top row) pYAP level for $N = 1$ large adhesion and $N = 9$ small adhesions with the same total area at different total counts of YAP, $n_{YAP} = 150, 250, 400, 800$. Increasing the YAP concentration reduces the effect of adhesion spatial distribution on pYAP level. Reducing the diffusion rate increases the effect of the spatial distribution of adhesions on the pYAP ratio (see for example the results for $n_{YAP} = 800$ for $D = 0.8$ *and* $0.5\ \mu m^2/s$). (Bottom row) The pYAP ratio, defined as pYAP divided by the total YAP, decreases as YAP concentration increases. This reduction is due to a higher number of YAP per adhesion site and limited available binding sites for YAP at higher concentrations. For other parameters see table S1.

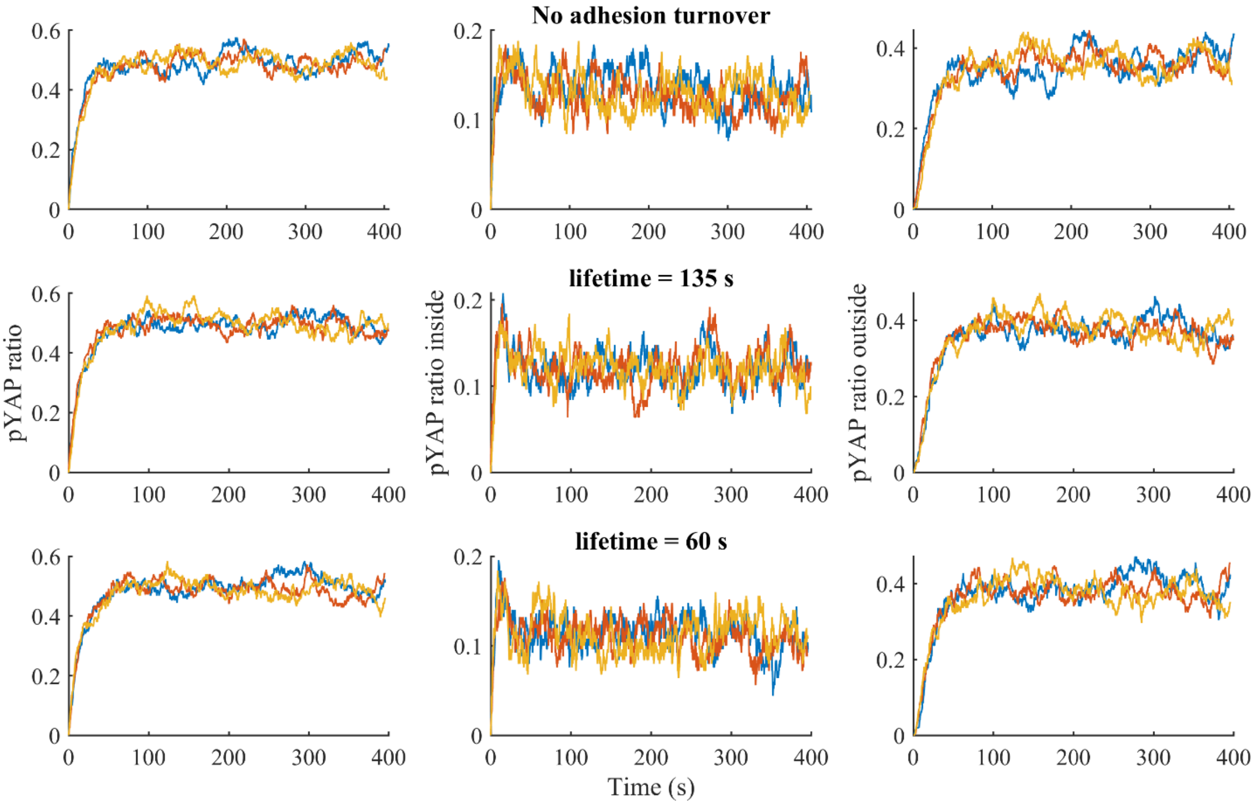

Figure S8*:* Temporal evolution of total pYAP ratio, pYAP ratio within the adhesions, and pYAP ratio outside the adhesions for nine small adhesions $N = 9$ using $R_{u,pYAP} = 0.1\ s^{-1}$, $R_{deph} = 0.035\ s^{-1}$: (Top row) no adhesion turnover, (Middle row) lifetime of 135 s, and (Bottom row) lifetime of 60 s*.* For other model parameters see table S3.

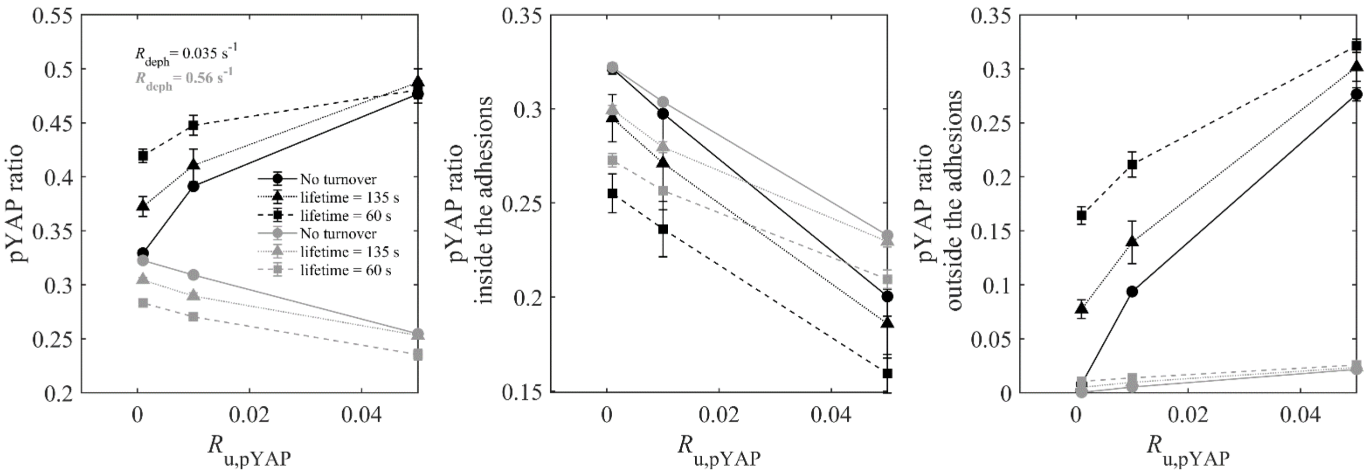

Figure S9*:* Zoomed-in panels from fig. 4 for $R_{u,pYAP} = 0.001 - 0.05\ s^{-1}$.

The total pYAP ratio, pYAP ratio within the adhesions, and pYAP ratio outside the adhesions for nine small adhesions $N = 9$ for three cases: no adhesion turnover, and lifetimes of 60 and 135 seconds for

two different dephosphorylation rates: $R_{dep} = 0.035\, s^{-1}$ (similar to Figs. 2,3) (black), and $R_{deph} = 0.56\, s^{-1}$ (gray). For the full parameter set see table S3.

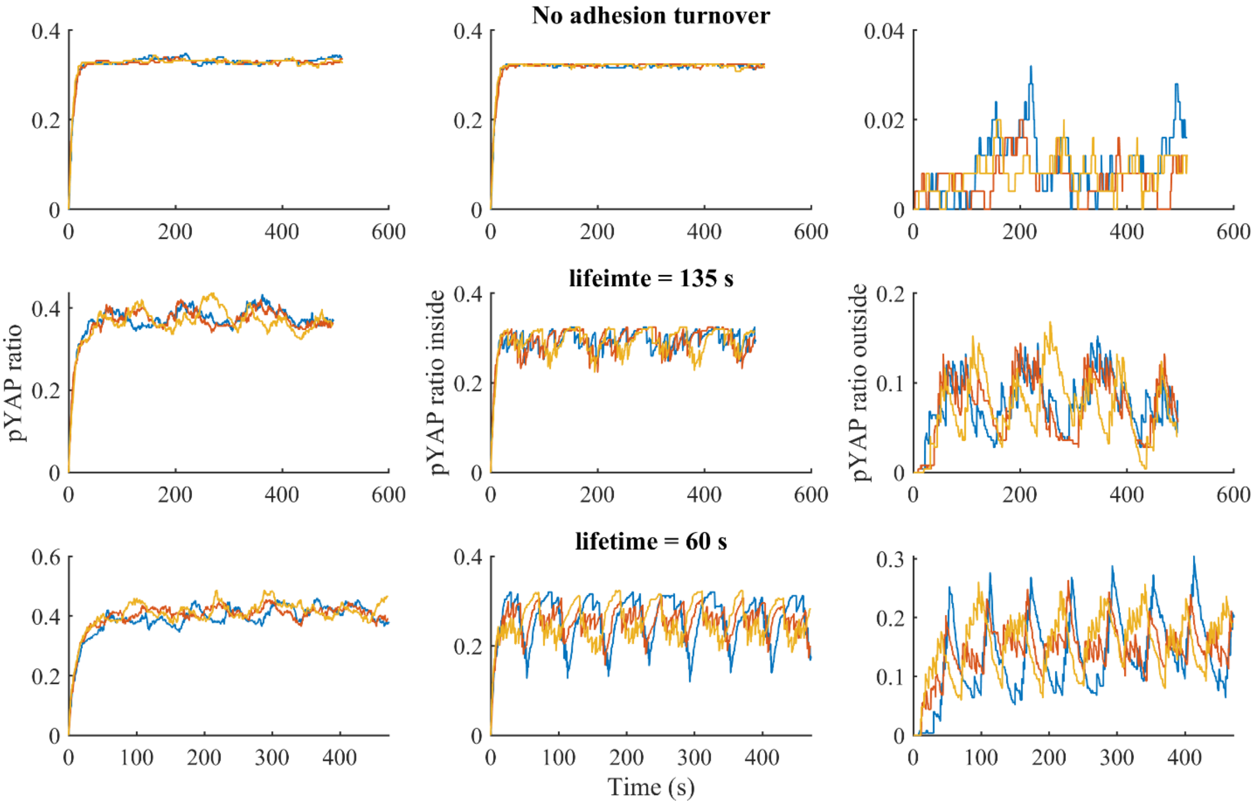

Figure S10: Temporal evolution of total pYAP ratio, pYAP ratio within the adhesions, and pYAP ratio outside the adhesions for nine small adhesions $N = 9$ using $R_{u,pYAP} = 0.001\, s^{-1}$, $R_{deph} = 0.035\, s^{-1}$: (Top row) no adhesion turnover, (Middle row) lifetime of 135 s, and (Bottom row) lifetime of 60 s. For other model parameters see table S3

Supplemental tables

Table S1: Parameter set and model variables used in Fig. 2.

Diffusion rate	D	0.8- 19 $\mu m^2/s$
YAP-adhesion binding rate	R_b	50 s^{-1}
YAP phosphorylation rate (after binding to the adhesion)	R_p	200 s^{-1}
YAP-adhesion unbinding rate	$R_{u,YAP}$	0.1 s^{-1}
pYAP-adhesion unbinding rate	$R_{u,pYAP}$	0.1 s^{-1}
Dephosphorylation rate	R_{deph}	0.035 s^{-1}
Node spacing	l	0.2 μm
Number of YAP	n_{YAP}	250
Number of adhesions	N	1,2,4,9
Lifetime	lifetime	N.A.
Number of binding sites	Number of binding sites	81
Adhesion size	Adhesion size	0.36-3.24 μm^2

Table S2: Parameter set and model variables used in Fig. 3.

Diffusion rate	D	0.8 $\mu m^2/s$
YAP-adhesion binding rate	R_b	5-100 s^{-1}
YAP phosphorylation rate (after binding to the adhesion)	R_p	200 s^{-1}
YAP-adhesion unbinding rate	$R_{u,YAP}$	0.1-0.2 s^{-1}
pYAP-adhesion unbinding rate	$R_{u,pYAP}$	0.1 s^{-1}
Dephosphorylation rate	R_{deph}	0.035 s^{-1}
Node spacing	l	0.2 μm
Number of YAP	n_{YAP}	250
Number of adhesions	N	1,9
Lifetime	lifetime	N.A.
Number of binding sites	Number of binding sites	81
Adhesion size	Adhesion size	0.36-3.24 μm^2

Table S3: Parameter set and model variables used in Fig. 4.

Diffusion rate	D	0.8 $\mu m^2/s$
YAP-adhesion binding rate	R_b	50 s^{-1}
YAP phosphorylation rate (after binding to the adhesion)	R_p	200 s^{-1}
YAP-adhesion unbinding rate	$R_{u,YAP}$	0.1 s^{-1}
pYAP-adhesion unbinding rate	$R_{u,pYAP}$	0.001-0.2 s^{-1}
Dephosphorylation rate	R_{deph}	0.035-0.56 s^{-1}
Node spacing	l	0.2 μm
Number of YAP	n_{YAP}	250
Number of adhesions	N	9
Lifetime	lifetime	60 and 135 s
Number of binding sites	Number of binding sites	81
Adhesion size	Adhesion size	0.36 μm^2

Table S4: Significance analysis for Fig. 2c and Fig. 2d. To calculate p-values we used two-sample t-test in MATLAB.

Figure 2c		Figure 2d	
Pairs	p-value	Pairs ($D = 0.8\ \mu m^2/s$)	p-value
Cases 1, 2	0.1004	$N = 1,2$	0.0148
Cases 1, 3	0.0154	$N = 1,4$	0.0006
Cases 1, 4	0.0006	$N = 1,9$	0.0006
Cases 2, 3	0.0423	$N = 2,4$	0.0007
Cases 2, 4	0.0008	$N = 2,9$	0.0011
Cases 3, 4	0.0330	$N = 4,9$	0.0141

Table S5: Fitting parameters for Fig. 3 (bottom panel).

Fitting function: $pYAP = k1R_b/(k2 + R_b)$		
Cases	$k1$	$k2$
$N = 1, D = 0.8\ \mu m^2/s$	101.3	5.4
$N = 9, D = 0.8\ \mu m^2/s$	143.7	7.1
$N = 1, D = 19\ \mu m^2/s$	210.6	12.1
$N = 9, D = 19\ \mu m^2/s$	213.7	11
